# Enhanced antiviral defense against begomoviral infection in *Nicotiana benthamiana* through strategic utilization of fluorescent carbon quantum dots to activate plant immunity

**DOI:** 10.1186/s12951-024-02994-4

**Published:** 2024-11-14

**Authors:** Tahir Farooq, Muhammad Dilshad Hussain, Yuan Wang, Ali Kamran, Muhammad Umar, Yafei Tang, Zifu He, Xiaoman She

**Affiliations:** 1 Plant Protection Research Institute and Guangdong Provincial Key Laboratory of High Technology for Plant Protection, Guangdong Academy of Agricultural Sciences, Guangzhou, 510640 P. R. China; 2 Key Laboratory of Agricultural Microbiology, College of Agriculture, Guizhou University, Guiyang, 550025 P. R. China; 3 New Town Research Laboratories, Tasmanian Institute of Agriculture, University of Tasmania, 13 St. Johns Avenue, New Town, Hobart, TAS 7008 Australia

**Keywords:** CQDs, Begomovirus, CLCuMuV, Photosynthesis, RNA-seq, Plant immunity, Alternative splicing

## Abstract

**Background:**

Owing to their unique physiochemical properties, low toxicity, antipathogenic effects and tunability, fluorescent carbon quantum dots (CQDs) represent a new generation of carbon-based nanomaterials. Despite the mounting research on the efficacy of CQDs against resilient plant pathogens, their potential ability to mitigate viral pathogens and the underlying molecular mechanism(s) remain understudied. In this study, we optimized the CQDs to maximize their antiviral effects against a highly pathogenic *Begomovirus* (cotton leaf curl Multan virus, CLCuMuV) and elucidated the mechanistic pathways associated with CQDs-mediated viral inhibition. To fine-tune the CQDs-induced antiviral effects against CLCuMuV and investigate the underlying molecular mechanisms,we used HR-TEM, XRD, FT-IR, XPS, and UV‒Vis spectrophotometry to characterize the CQDs. SPAD and FluorCam were used for physiological and photosynthetic performance analysis. Transcriptome, RT‒qPCR, integrated bioinformatics and molecular biology were employed to investigate gene expression, viral quantification and data validation.

**Results:**

The application of fluorescent, hexagonal crystalline, UV-absorptive and water-soluble CQDs (0.01 mg/ml) significantly reduced the CLCuMuV titer and mitigated viral symptoms in *N. benthamiana* at the early (5 dpi) and late (20 dpi) stages of infection. CQDs significantly increased the morphophysiological properties, relative chlorophyll contents and photosynthetic (*Fv/Fm*, *QY_max*, *NPQ* and *Rfd*) performance of the CLCuMuV-infected plants. While CLCuMuV infection disrupted plant immunity, the CQDs improved the antiviral defense response by regulating important immunity-related genes involved in endocytosis/necroptosis, Tam3-transposase, the ABC transporter/sphingolipid signaling pathway and serine/threonine protein kinase activities. CQDs potentially triggered TSS and TTS alternative splicing events in CLCuMuV-infected plants.

**Conclusions:**

Overall, these findings underscore the antiviral potential of CQDs, their impact on plant resilience, and their ability to modulate gene expression in response to viral stress. This study’s molecular insights provide a foundation for further research on nanomaterial applications in plant virology and crop protection, emphasizing the promising role of CQDs in enhancing plant health and combating viral infections.

**Graphical Abstract:**

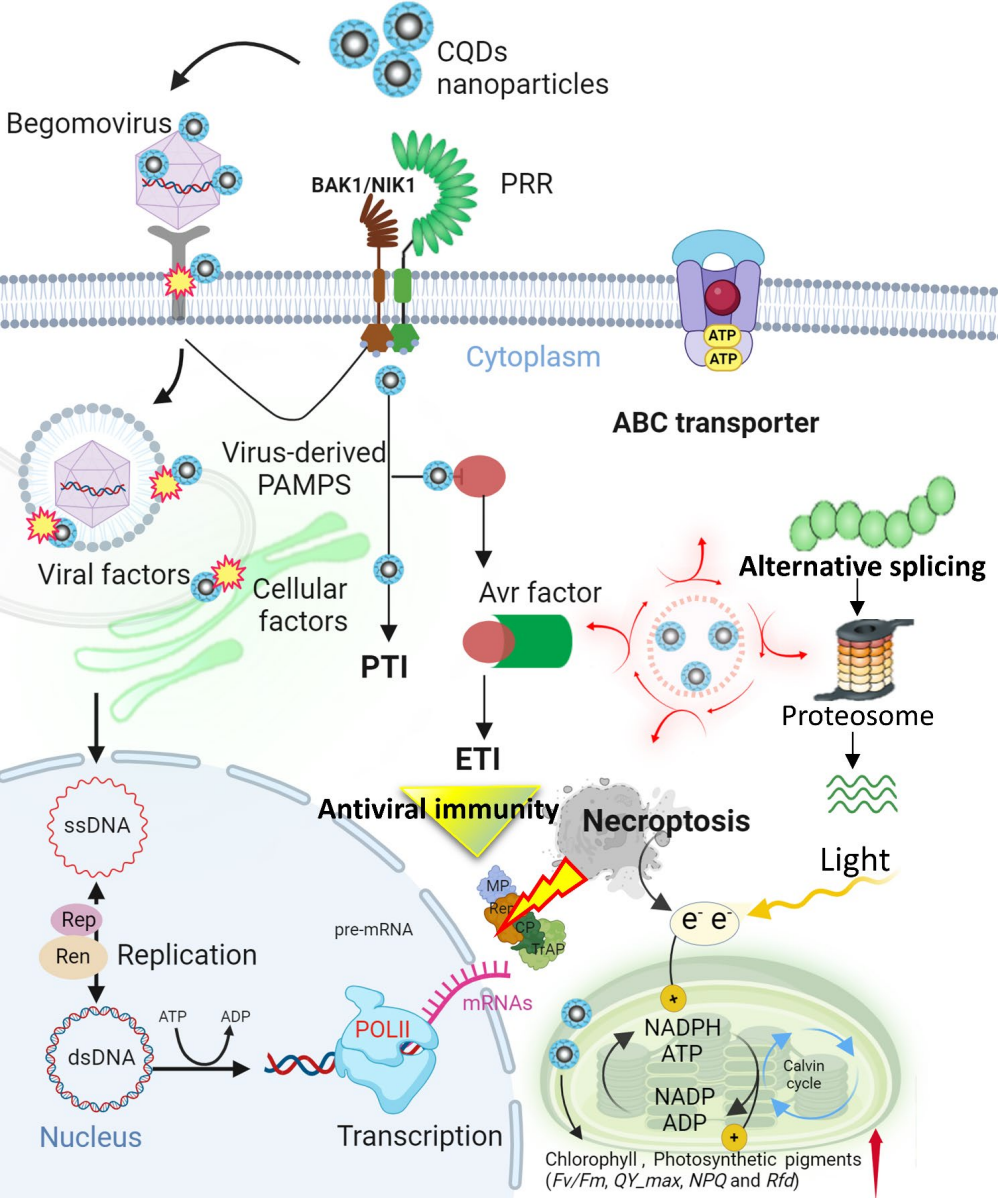

**Supplementary Information:**

The online version contains supplementary material available at 10.1186/s12951-024-02994-4.

## Background

Begomoviruses are a major group of potentially damaging single-stranded DNA (ssDNA) plant viruses that are transmitted exclusively by the insect vector whitefly (*Bemisia tabaci*). They have a wide host range and cause serious looming threats to fiber and food crops, thereby contributing to substantial risks to global food security [1, 2]. The *cotton leaf curl Multan virus* (CLCuMuV) is one of the most dominant species of *Begomoviruses* (family *Geminiviridae*) and a major constraint on global cotton production [3, 4]. To manage this potentially damaging virus along with other pathogenic phytoviruses, it is imperative to plan, design tests, and develop different antiviral strategies to ensure effective and sustainable plant disease management worldwide.

In recent decades, nanotechnology has emerged as an efficient and innovative technology with significant potential in combating plant pathogens (especially viruses) to achieve sustainable disease management [5, 6]. Engineered nanoparticles (NPs) play significant roles in increasing plant immune responses and inhibiting virus replication. For example, zinc oxide NPs (ZnONPs) and silica NPs (SiO_2_NPs) directly interact with the viral capsid protein (CP), inducing structural disruption, viral aggregation, and rapid deactivation of tobacco mosaic virus (TMV) at the site of infection [7]. Furthermore, these NPs markedly increase plant defense responses, especially reactive oxygen species (ROS) accumulation, catalase and/or peroxidase activity, and the expression of systemic resistance-related genes (*PR1* and *PR2*) [7]. Similarly, iron NPs (Fe_2_O_3_ NPs) suppress infection by turnip mosaic virus (TuMV) [8], and silver NPs (AgNPs) can bind successfully to the CPs of tomato mosaic virus (ToMV) and potato virus Y (PVY) and inhibit their systemic infection [9]. Gold NPs (AuNPs) can damage virus-like particles (VLPs) associated with barley yellow dwarf virus-PAV (BYDV-PAV) [10]. Carbon-based nanomaterials (CNMs) and C-based nanotubes (CNTs) suppress the systemic infection of TMV through the augmentation of plant immunity by enhancing photosynthetic performance and ultimately inducing viral resistance in the host [11]. However, the applications of these NPs are limited due to their toxicity and/or cytotoxicity, the inertness of the starting material, colloidal instability, and complicated synthesis processes.

Recently, biomedical and nanotechnological research in nanomedicine and nanotherapeutics has intensified in pursuit of novel sustained therapies with improved efficacy and a targeted approach [12]. Carbon quantum dots (CQDs) are 0-dimensional and novel small (2–8 nm) carbon-based nanoparticles with several advantageous characteristics (excellent biocompatibility, high sustainability, low/no toxicity, and designed biocompatibility under physiological settings) [12–14]. Given their diverse and innovative properties, in addition to being antibacterial and antifungal, CQDs have gained much attention for their ability to investigate and curb devastating viral diseases (coronavirus, norovirus, flaviviruses, arterivirus, and herpesvirus) in the contemporary era [14, 15]. Notably, CQDs are versatile antiviral agents that combat virus entry and replication, dynamically interacting with various stages of viral infection (attachment, penetration, replication, and propagation) [14, 16]. Such CQDs- and cadmium telluride quantum dots (CdTe QDs)-based ratiometric fluorescence biosensors are involved in the detection of dsDNA human immunodeficiency virus (HIV) [17], and boronic acid-modified CQDs inhibit HIV infection by interacting with gp120 and thereby disrupting subsequent interactions with target cells [18]. Similarly, boronic acid- or amine-designed carbon nanodots (C-dots) bind to the cell membrane and block virus‒host interactions to prevent herpes simplex virus type 1 (HSV-1) infection [19]. In addition to exhibiting antibacterial activity, polyamine CQDs also directly interact with the virion envelope of white spot syndrome virus (WSSV) to combat viral infection [20]. Triazole-based CQDs block viral replication enzymes such as helicases and 3-chymotrypsin-like protease (3CLpro) and potentially inhibit human coronaviruses [21]. Currently, curcumin cationic carbon dots (CCM-CDs) significantly combat COVID-19 via prevention of viral entry, generation of ROS, budding, and synthesis of negative-strand RNA [16, 22].

In the case of plant viruses, CQDs have been used only for dsRNA delivery and antiviral protection until recently. Xu et al. (2023) evaluated CQDs with other NPs, amine-functionalized silica nanopowder (ASNP) and chitosan quaternary ammonium salt (CQAS) and reported that CQDs are efficient tools for dsRNA delivery and the suppression of systemic infection by potato virus Y [23]. Unfortunately, research on the dedicated application of CQDs to combat plant viruses is limited. Considering the growing interest in the multifaceted antiviral properties of CQDs, precise characterization and fine-tuning/optimization are needed before their application. The aim of the present study was to fine-tune the CQDs-induced antiviral effects against a DNA plant virus (CLCuMuV) and to investigate the underlying molecular mechanisms. Preliminarily, we tested the effectiveness of different concentrations of CQDs against viral infection. Furthermore, we performed comprehensive transcriptomic profiling of *N. benthamiana* plants in response to viral infection and CQDs treatment at the early and late stages of viral infection. The findings of this study reveal the molecular mechanism(s) associated with the antiviral activity of CQDs. This will open new avenues for the design, synthesis, and application of CQDs-based antiviral strategies for the sustainable management of viral diseases.

## Materials and methods

### Source and maintenance of plants, virus inoculum and agroinoculation

Wild-type (WT) *Nicotiana benthamiana* plants were maintained in a substrate mixture of vermiculite, perlite, black soil and artificial soil (2:2:2:1) at ~ 60% relative humidity (RH), with a 14-h light and 10-h dark photoperiod and a temperature ranging from 25 to 27 °C. At the 5–7 fully expanded leaf stage, *N. benthamiana* plants were infiltrated with CLCuMuV (DNA-A + β) following a previously established protocol [24]. Briefly, *Agrobacterium tumefaciens* (strain GV3101) harboring CLCuMuV DNA-A (KP762786) and associated betasatellite (KP762787) were grown overnight in liquid broth supplemented with appropriate antibiotics to reach an OD_600_ = 1. The bacterial constructs containing CLCuMuV DNA-A and betasatellite were suspended in infiltration buffer (10 mM MES, 10 mM MgCl_2_, and 150 µM acetosyringone, pH 5.6), mixed at a 1:1 ratio, and used to challenge the lower epidermis of *N. benthamiana* plants with a 1 ml needleless syringe. The plants were regularly observed for the development of symptoms, and the presence of the virus was confirmed via the specific primers CLCuMuV-CL F/R & CLCuMuB-betaF/R (Supplementary Table 1). In all the experiments, CN represents the untreated negative/healthy control, CP corresponds to the virus-infected positive control, QD represents the CQDs-treated, virus-free plants, and QV corresponds to the virus-infected and CQDs-treated *N. benthamiana* plants. The numbers 5 and 20 represent the days post-inoculation (dpi). The “CLCuMuV” represents co-infection by DNA-A + betasatellite unless otherwise specified.

### Preparation, characterization and application of CQDs

The fluorescent, water-soluble CQDs were synthesized via hydrothermal treatment with malic acid [25]. The amine functionalization of the CQDs was achieved via the use of ethylenediamine, and cysteine was used as the capping/reducing agent. To analyze the physiochemical properties of the obtained CQDs, systematic characterization was performed via high-resolution transmission electron microscopy (HR-TEM) (FEI, Talos F200X). To obtain information on the CQDs particle size, crystal structure and phase purity, X-ray diffraction (XRD) was performed via a Bruker D8 ADVANCE X-ray diffractometer (40 mA, 40 kV, 10° -90°, 5°/min). Fourier transform infrared (FT-IR) spectrum analysis was performed to analyze the presence of functional groups associated with the CQDs. For FT-IR analysis, a Nicolet iS 10 FTIR spectrometer was used with a spectral range of 400–4000 cm^− 1^. To further confirm the FT-IR assignments, quantitative spectroscopic measurements were taken via X-ray photoelectron spectroscopy (XPS) using a Thermo ESCALAB 250XI. The XPS-specific parameters were monochromatic Al Kα (hv = 1486.6 eV) with a power of 150 W, 650 μm beam spot, voltage of 14.8 kV, current of 1.6 A, and potential correction using contaminated carbon C1s = 284.8 eV for correction. The steady-state photoluminescence (PL) intensity of the CQDs was characterized via an Edinburgh FLS980 instrument. Absorption spectra were analyzed via a UV‒Vis spectrophotometer (UV‒2600). In a preliminary experiment, various concentrations of CQDs were prepared and tested for their efficacy against CLCuMuV at different time points (Supplementary Fig. 1). Based on the initial results (Supplementary Fig. 2), a 0.01 mg/ml concentration was used in the current study. The *N. benthamiana* plants were foliar sprayed with CQDs (0.01 mg/ml) 48 h before agroinfiltration.

### Estimation of morphological, physiological and disease-related parameters

To assess plant growth, development and physiological performance in response to CLCuMuV and CQDs treatment, qualitative plant growth parameters, including leaf area and fresh weight, were measured at 5 and 20 dpi to obtain representative data for the early and late stages of virus infection, respectively. The infection percentage was calculated by assessing the number of symptomatic/PCR-positive plants against the total number of agroinfiltrated *N. benthamiana* plants. The leaf samples from all the treatments were either fresh or preserved at -80 °C for downstream applications.

The relative chlorophyll contents (*Chla* and *Chlb*) were measured in situ via a hand-held soil plant analysis development (SPAD)-502-Plus chlorophyll meter (Konica Minolta, Inc., Osaka, Japan). The SPAD system analyzes the level of leaf greenness coupled with the interaction of incident light and thylakoid chlorophyll. At least 3 points (15–35 mm away from the leaf midrib) on each leaf were selected for SPAD readings at 650/940 nm. As previously described [11] the key parameters associated with photosynthetic performance (*Fv/Fm*. *QY-Lss*, *NPQ* and *Rfd-Lss*) were measured via a fluorometer (Fluorcam 800 MF, equipped with FC-800D/355 − 15 and 735 nm LEDs). The software (Fluorcam 7.0) was used to process and analyze the images.

### cDNA library preparation, transcriptome profiling, assembly and analyses

Approximately 1.5 µg of RNA from each sample was processed to construct paired-end cDNA libraries. The cDNA libraries were generated via the ABclonal mRNA-seq Lib Prep Kit (ABclonal, China) following the provided protocol, and subsequently, the products were purified via the AMPure XP system according to the manufacturer’s protocol. The qualitative assessment of the purified products was performed via an Agilent Bioanalyzer 4150 system. The sequencing of these products was carried out in paired-end mode via the NovaSeq 6000/MGISEQ-T7 sequencing platform (Illumina BGI, China). After that, the low-quality reads and those with adapters or poly-N sequences were removed to obtain clean data. The data were filtered to obtain high-quality, paired-end reads that were aligned to the *N. benthamiana* reference genome (Niben-V.261). To count the number of reads mapped to *N. benthamiana* genes, HTSeq (v0.6.1) was employed in Python. The expected values of the transcripts per million (TPM) and fragments per kilobase per million mapped fragments (FPKM) were obtained by analyzing the faction of transcripts/cells and estimation of read counts mapped to the target gene and the relative gene length, respectively.

### Comparative statistical analyses between different samples

The analysis of DEGs between all comparative groups with and without CLCuMuV and CQDs treatment at 5 and 20 dpi was performed via the DESeq2 package in R as described previously [26]. For multiple testing, the false discovery rate (FDR) [27] was employed to adjust the raw *P value*s. The cutoff FC values of ≥ 2 and ≤-2 were used to identify the DEGs with an adjusted *P value* (Padj) of < 0.05. Principal component analysis (PCA) [28] was used to analyze the data variability between different comparative groups. Analysis of similarity (ANOSIM) [29] was employed as a nonparametric test to identify significant differences between groups. To calculate the distance between different samples/groups, hierarchical cluster tree analysis was performed via the Bray‒Curtis statistical algorithm in R’s Vegan package [30].

### DEG functional annotation and pathway enrichment analyses

To obtain comprehensive information about the functional classes and enrichment pathways of the candidate DEGs from key comparative groups, three databases were used: GO (Gene Ontology) (http://www.geneontology.org) [31], KEGG (Kyoto Encyclopedia of Genes and Genomes) (http://www.kegg.jp) [32] and KOG (euKaryotic Orthologous Groups) (https://www.ncbi.nlm.nih.gov/COG/).

### Identification of different patterns of alternate splicing

The genomic mapping results were assembled with StringTie [33] and then compared with known gene models via GffCompare [34] to discover new transcriptional regions. Variable splicing events were classified via ASprofile software [35] on the basis of the predicted gene model for each sample. In total, 12 alternative splicing (AS) types were identified and compared across different comparative groups at 5 and 20 dpi in response to CLCuMuV infection and CQDs treatment. These AS types included alternative 5’ first exon (TSS), alternative 3’ last exon (TTS), skipped exon (SKIP), approximate SKIP (XSKIP), multiexon SKIP (MSKIP), approximate MSKIP (XMSKIP), intron retention (IR), approximate IR (XIR), multi-IR (MIR), approximate MIR (XMIR), alternative exon ends (AE) and approximate AE (XAE).

### Quantification and validation of candidate DEGs via RT‒qPCR

To validate the RNA-seq results, the mRNA expression profiles of 16 candidate DEGs (Supplementary Table 2) were quantified via reverse transcription‒quantitative polymerase chain reaction (RT‒qPCR) and normalized against glyceraldehyde 3-phosphate dehydrogenase (*GAPDH*) (GenBank accession: JQ256517) and protein phosphatase 2 A (*PP2A*) (GenBank accession: MF996339) as the internal control genes. The selected genes were categorized into three groups on the basis of these criteria: (i) their putative association with plant defense/immunity-related pathways in response to CQDs treatment and CLCuMuV infection, (ii) their significantly high or low expression levels among key comparative groups, and (iii) their randomly selected DEGs [36]. The RNA extraction was performed as described above, and cDNA synthesis was carried out via the PrimeScript™ II 1st strand cDNA Synthesis Kit (TAKARA) following the manufacturer’s protocol. The RT‒qPCR experiment was performed via TB Green^®^*Premix Ex Taq*™ II (Tli RNaseHPlus) (Takara Bio, Inc.) according to the provided instructions. To calculate the relative mRNA expression, the 2^−ΔΔCT^ method was employed as reported previously [37]. For each treatment, a total of three biological and nine technical replicates were included. A list of primers used for RT‒qPCR analysis is given in Supplementary Table 3.

### Statistical analyses

For the leaf fresh weight and leaf area data, the statistically significant differences among the different treatment groups were analyzed via the Holm‒Sidak method, with α = 0.05. Bartlett’s and Shapiro‒Wilk’s tests were performed to determine the homogeneity of variance and normal distribution of the data before they were tested with ANOVA. The relative abundances of candidate DEGs and CLCuMuV in *N. benthamiana* plants were compared via an independent *t* test. The statistically significant differences among different comparison groups were designated on the basis of *P* values (*P < 0.05, **P < 0.01, ***P < 0.001).

## Results

### Photology, morphology and structural characterization of the CQDs

**Fig. 1:**
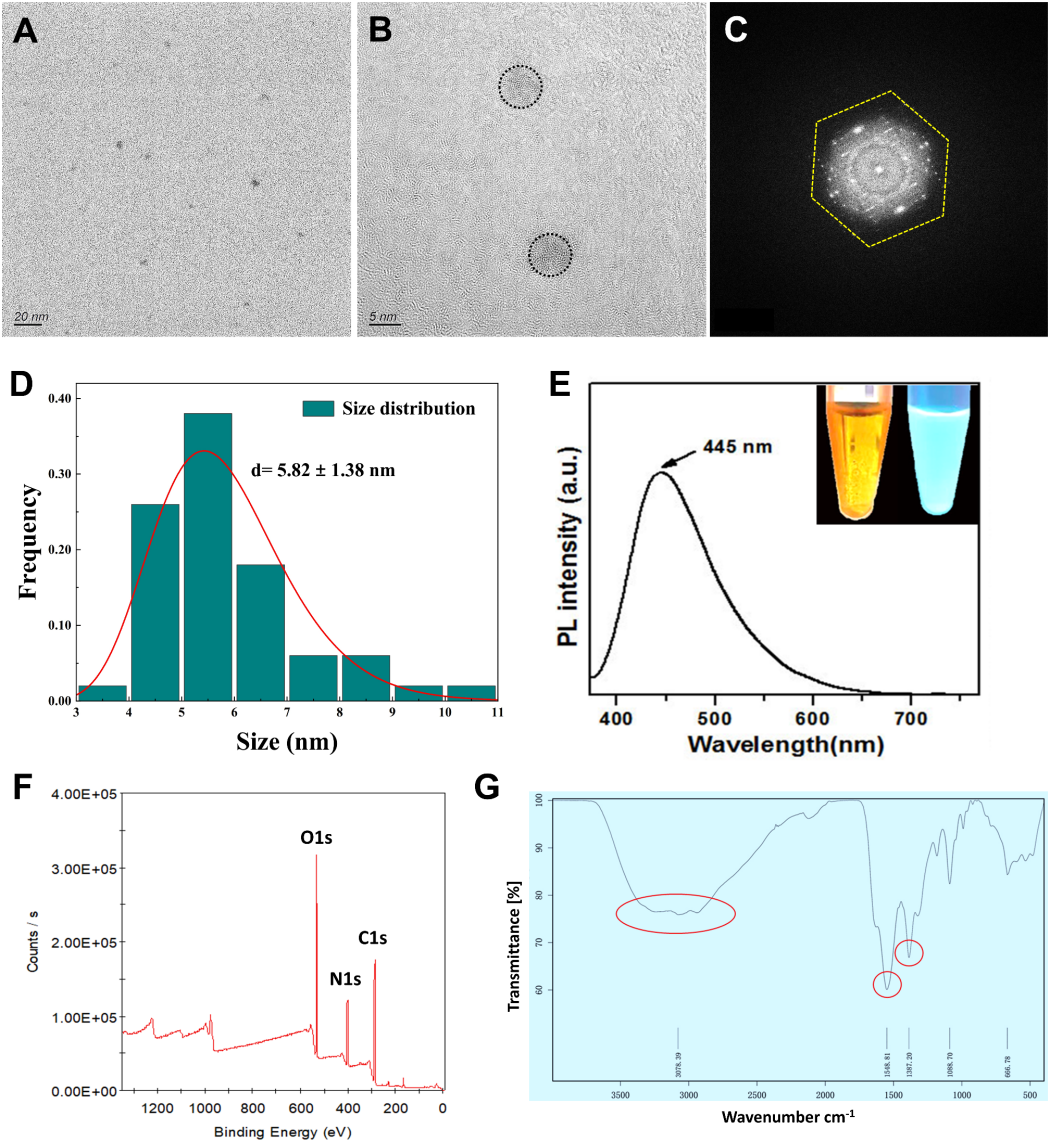
Morphological and structural peculiarities of CQDs. (**A**-**B**) High-resolution transmission electron microscopy (HR-TEM), (**C**) hexagonal crystalline structure observed via fast Fourier transform (FFT) test, (**D**) particle distribution analysis, (**E**) photoluminescence (PL) intensity and UV‒Vis spectrophotometer analyses, (**F**) X-ray photoelectron spectroscopy (XPS) testing, and (**G**) Fourier transform infrared (FT-IR) spectrum analysis.

A series of analyses, including HR-TEM, FTIR, XRD and UV‒Vis spectrophotometry, were used to characterize the fluorescent CQDs. HR-TEM observations revealed that the CQDs had a uniform, spherical morphology and monodispersity (Fig. 1A-B). The CQDs exhibited a hexagonal single-crystalline structure (Fig. 1C) and a narrow average particle size distribution with a mean size of 5.82 ± 1.38 nm (Fig. 1D). The CQDs appeared cyan blue under ultraviolet (UV) light and displayed a photoluminescent excitation wavelength of 445 nm (Fig. 1E). Elemental composition analysis via photoelectron spectroscopy revealed the presence of representative binding energy peaks at 531, 400 and 285 eV attributed to O1s, N1s and C1s, respectively (Fig. 1F). This result revealed that the CQDs were mainly composed of C, N and O. Next, FT-IR was employed to observe the surface state of the CQDs. As shown in Fig. 1G, a strong absorption band ranging between 3000 and 3500 cm^− 1^ was observed, which corresponded to the stretching vibrations of O-H and N-H. The sharp peak at 3078.39 cm^− 1^ was attributed to O-H. Additionally, the representative absorption peaks centered at 1548.81 and 1387.20 cm^− 1^ were attributed to the stretching vibrations of N-H and C-NH-C, respectively (Fig. 1G). Finally, the zeta potential of -18.4 mV corresponds to the negatively charged surface of the CQDs, which is attributed to the presence of many functional amino and hydroxyl groups.

### Early CQDs application significantly reduced CLCuMuV accumulation and the development of disease symptoms

**Fig. 2:**
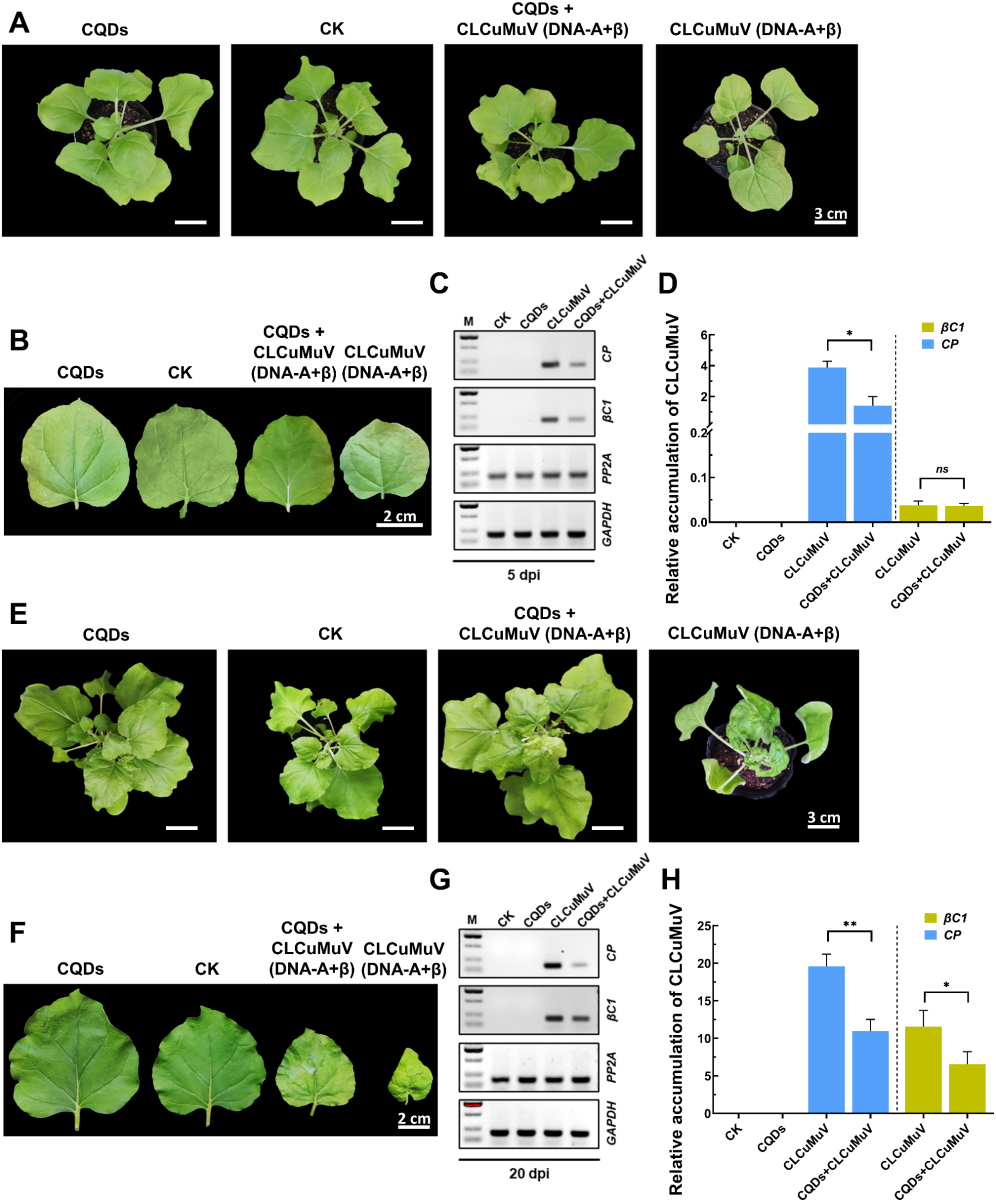
CLCuMuV symptoms and viral abundance at the early (5 dpi) and late (20 dpi) stages of infection. (**A**) Representative images of *N. benthamiana* plants from groups subjected to different treatments, including uninfected healthy (CK), CQDs-treated (CQDs), virus-infected (CLCuMuV) and CQDS-treated + virus-infected (CQDs + CLCuMuV) groups, at 5 dpi. (**B**) Leaf morphology of *N. benthamiana* in the four comparative groups at 5 dpi. (**C**) Semiquantitative RT‒PCR of representative samples corresponding to each comparative group at 5 dpi. *PP2A* and *GAPDH* were used as internal controls. M represents the GL 2,000 DNA marker, while each band represents a specific 200 bp amplicon corresponding to the tested genes. (**D**) Relative abundance of CLCuMuV transcripts quantified via RT‒qPCR at 5 dpi. (**E**) Comparison of the morphology of the healthy and CQDs-treated groups and the development of viral symptoms among the CLCuMuV (DNA-A + betasatellite)-infected plants with and without CQDs treatment at 20 dpi. (**F**) Leaf morphology of healthy and CQDs-treated plants and representative leaves showing typical CLCuMuV symptoms. (**G**) Semiquantitative RT‒PCR of representative samples corresponding to each comparative group at 20 dpi. *PP2A* and *GAPDH* were used as internal controls. M represents the GL 2,000 DNA marker, while each band represents a specific 200 bp amplicon corresponding to the tested genes. (**H**) Relative quantification of CLCuMuV at 20 dpi. The statistically significant differences among different comparison groups were designated on the basis of *P values* (*P<0.05, **P<0.01)

The agroinfiltrated plants with and without CQDs treatment were regularly monitored and compared with those in the control groups. At 5 dpi, the CLCuMuV-treated plants presented typical viral symptoms of leaf curling, mottling and mosaic in the apical/systemic leaves (Fig. 2A-B). Similarly, a pattern of viral symptom development was observed in the CLCuMuV-treated plants with CQDs application, although the degree of symptom intensity was slightly lower than that in the plants without CQDs treatment (Fig. 2A). As viral symptom expression is directly correlated with the viral titer in agroinfiltrated plants, we also analyzed the relative accumulation of CLCuMuV transcripts in all the plants. The positive PCR results revealed a specific 200 bp amplicon corresponding to the *CP* and *βC1* genes, confirming the presence of CLCuMuV in the plants at 5 dpi (Fig. 2C). RT‒qPCR analysis of CLCuMuV-infected plants with and without CQDs treatment further provided insights into the relative abundance of viral transcripts. The results of the quantitative gene expression analysis revealed that the relative accumulation of the coat protein (CP) of CLCuMuV was 64.17% lower in the plants that were treated with the CQDs (Fig. 2D). Similarly, viral symptoms and relative viral abundance were measured at 20 dpi, which revealed strong viral symptoms in the CLCuMuV-infected plants. These plants presented severe leaf curling, mosaic, vein thickening and yellowing symptoms accompanied by stunted growth (Fig. 2E). Surprisingly, the CQDs-treated plants retained a phenotype that strongly resembled that of the healthy control (Fig. 2F). The results of semiquantitative RT‒PCR indicated a relatively high abundance of the viral *CP* and *βC1* genes in the plants without CQDs treatment, while the viral titer was noticeably reduced in response to CQDs application (Fig. 2G). Although the CQDs-treated plants presented mild viral symptoms, the relative accumulation of CLCuMuV was significantly (*P* < 0.01) lower than that in the CLCuMuV-infected plants without CQDs treatment (Fig. 2H). Overall, CQDs application significantly reduced the viral titer and the development of CLCuMuV symptoms in agroinfiltrated plants and augmented the overall plant morphology despite the presence of viral infection.

### CQDs treatment augmented the morphophysiological properties and photosynthetic performance of CLCuMuV-infected *N. benthamiana*

**Fig. 3:**
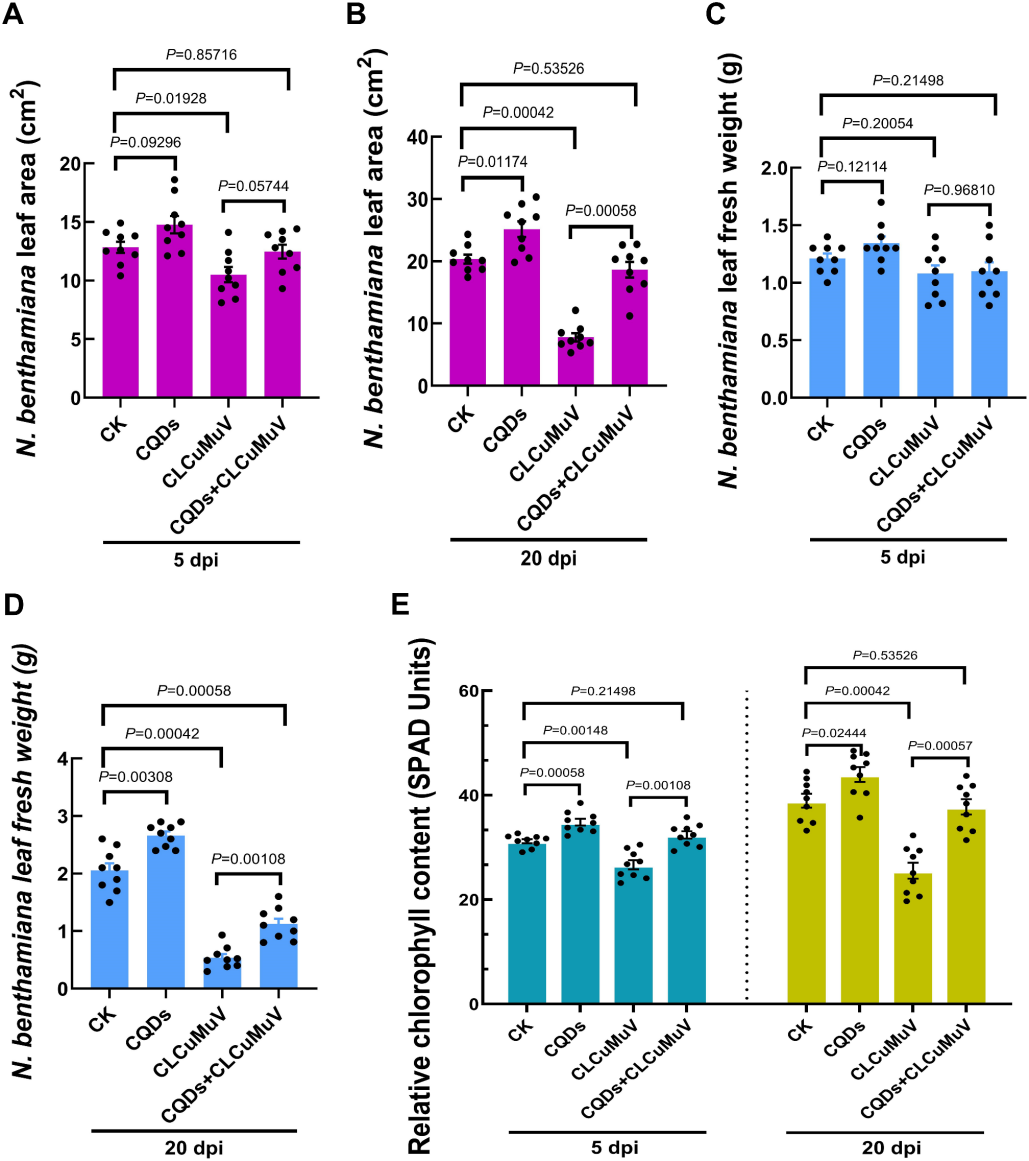
Estimation of morphophysiological parameters, chlorophyll measurements and photosynthetic performance among various comparative groups. (**A**-**B**) Leaf areas of healthy, CQDs-treated and CLCuMuV-infected *N. benthamiana* plants with and without CQDs treatment at 5 and 20 dpi. (**C**-**D**) Fresh weight of *N. benthamiana* leaves corresponding to the negative (CK)-positive (CLCuMuV) control and CQDs-treated groups with and without viral infection at 5 and 20 dpi. (**E**) Relative estimation of the chlorophyll content. A *P value* < 0.05 denotes a significant difference among the comparative groups

Given that viral infection is strongly correlated with impaired plant morphology and physiological performance, we sought to analyze and compare the effects of CQDs treatment on the morphophysiological properties and photosynthetic performance of CLCuMuV-infected plants. At 5 dpi, CQDs application (0.01 mg/ml) did not significantly (*P* = 0.09296) increase the leaf area compared with that of healthy plants (Fig. 3A). CLCuMuV infection significantly (*P* = 0.01928) reduced the leaf area by 18% compared with that of uninfected healthy plants. Notably, CQDs treatment of the CLCuMuV-infected plants increased the leaf area by 17.6%, although the difference was not significant (*P* = 0.05744) (Fig. 3A). The effect of CQDs application was prominent at 20 dpi, where it increased the biomass of the CLCuMuV-infected plants by 139%, resulting in a significant (*P* = 0.00058) difference in the leaf area compared with the 61% decrease in the leaf area of the CLCuMuV-infected plants. In general, regardless of CLCuMuV infection, at 0.01 mg/ml, the CQDs significantly (*P* = 0.01174) increased the leaf area of *N. benthamiana* by 23% (Fig. 3B). Furthermore, at 5 dpi, CQDs application increased the biomass of the uninfected plants by 11%, whereas this difference was not significant (*P* = 0.9681) in the case of the CLCuMuV-infected plants, where the CQDs treatment increased the fresh weight by only 1.85% (Fig. 3C). However, at 20 dpi, the effects of the CQDs on the CLCuMuV-infected plants were significant (*P* = 0.00108), where the fresh area of the virus-infected plants increased by 109% compared with that of the plants in which CLCuMuV infection significantly (*P* = 0.00042) reduced the fresh weight by 73% (Fig. 3D). The results of SPAD analysis revealed that CQDs treatment significantly increased the relative chlorophyll content among healthy plants by 11 (*P* = 0.00058) and 12.9% (*P* = 0.02444) at 5 and 20 dpi, respectively (Fig. 3E). The plants that were infected with CLCuMuV presented significantly lower chlorophyll contents, which were reduced by 14 (*P* = 0.00148) and 34% (*P* = 0.00042) at 5 and 20 dpi, respectively. However, in the presence of CLCuMuV infection, the CQDs significantly increased the chlorophyll content by 21% (*P* = 0.00108) and 47% (*P* = 0.00057) at 5 and 20 dpi, respectively (Fig. 3E).

**Fig. 4:**
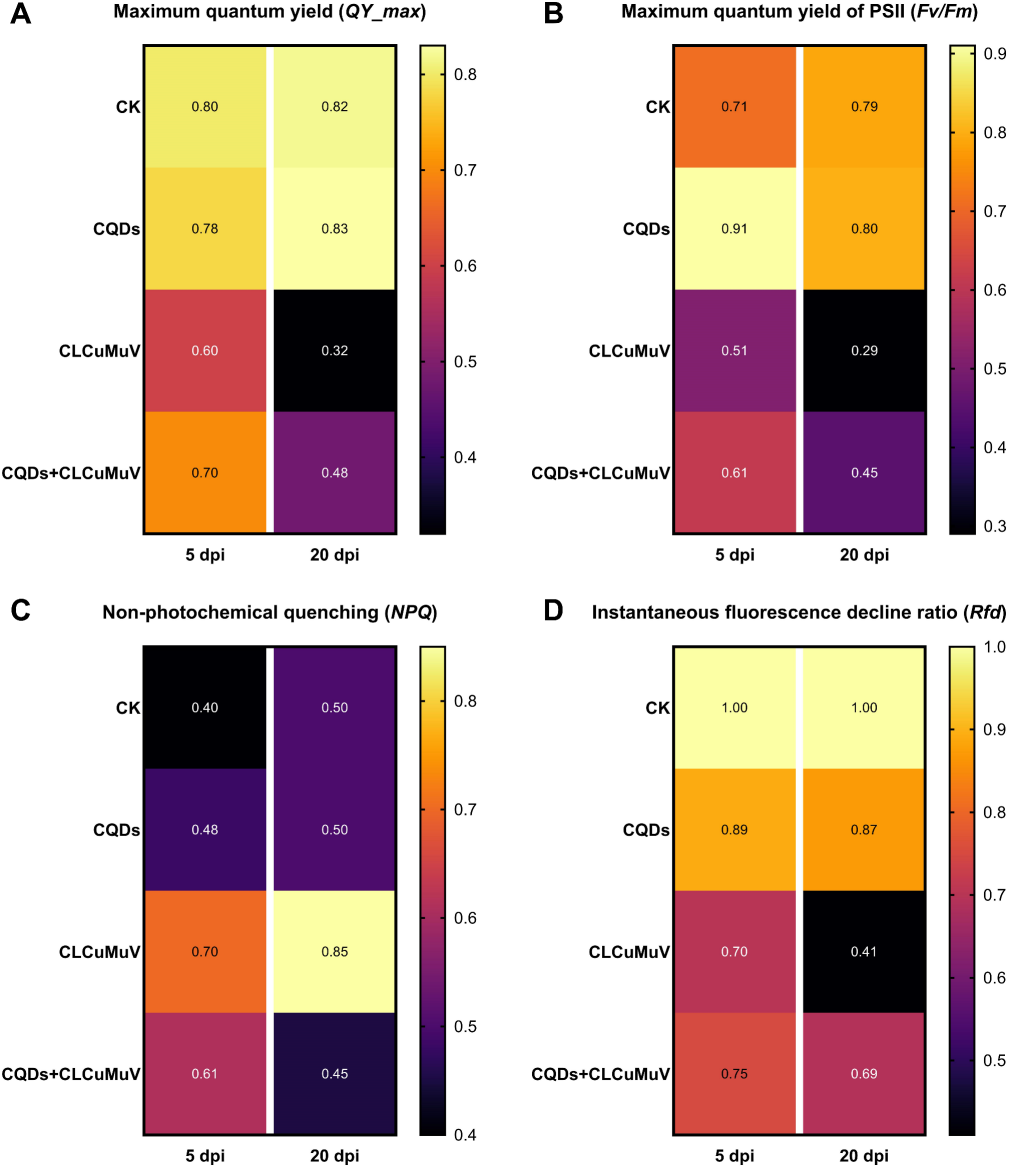
Comparison of key photosynthetic parameters among different treatments. (**A**) Relative variation in the photosynthetic efficiency/*QY*-max (**B**) maximum quantum yield of photosystem II/*Fv/Fm* (**C**) nonphotochemical quenching of absorbed energy/NQP and (**D**) chlorophyll fluorescence decline ratio/*Rfd* among healthy, virus-infected and CQDs-treated *N. benthamiana* plants in the presence and absence of CLCuMuV infection at 5 and 20 dpi

Next, to evaluate the photosynthetic performance of the CLCuMuV-infected plants in response to CQDs treatment, we analyzed several key chlorophyll fluorescence parameters, including *QY*-max, *Fv/Fm*, *NPQ* and *Rfd*. The results revealed that in the CQDs-treated plants, the steady-state levels of the maximum quantum yield of photosystem II (PSII) (*Fv/Fm* and *QY*-max) presented patterns of fluorescence similar to those of the uninfected healthy control plants at 5 and 20 dpi (Fig. 4A-B). However, in the absence of CQDs, CLCuMuV infection significantly impaired the photosynthetic machinery of the host plants, as reflected by reduced values of *Fv/Fm* and *QY*-max at 5 and 20 dpi (Fig. 4A-B). Strikingly, the effect of the CQDs in the CLCuMuV-infected plants was positively correlated with photosynthetic performance, as reflected by higher *Fv/Fm* and *QY*-max values at the early and late stages of infection (Fig. 4A-B). The *NPQ* values (which represent the biotic/abiotic stress level of the leaf tissues) were significantly greater among the CLCuMuV-infected plants at both 5 and 20 dpi, whereas the CQDs application appeared to eliminate the virus-induced stress in the host plants, as indicated by the lower *NPQ* values (Fig. 4C). Furthermore, the ratio of fluorescence decrease (*Rfd*) was greatly reduced among virus-infected plants at 5 and 20 dpi, highlighting that CLCuMuV-induced stress greatly reduced overall plant viability. Conversely, the plants treated with the CQDs were able to maintain relatively high values of *Rfd* despite the presence of viral infection, which indicated that the CQDs can help eliminate the damaging effect of viral infection (Fig. 4D). In conclusion, these results clearly demonstrate that CQDs application can significantly improve the morphophysiological and photosynthetic performance of virus-infected plants.

### Comparative transcriptome profiling of *N. benthamiana* plants at the early (5 dpi) and late (20 dpi) stages of viral infection, data quality and transcriptome assembly

**Fig. 5:**
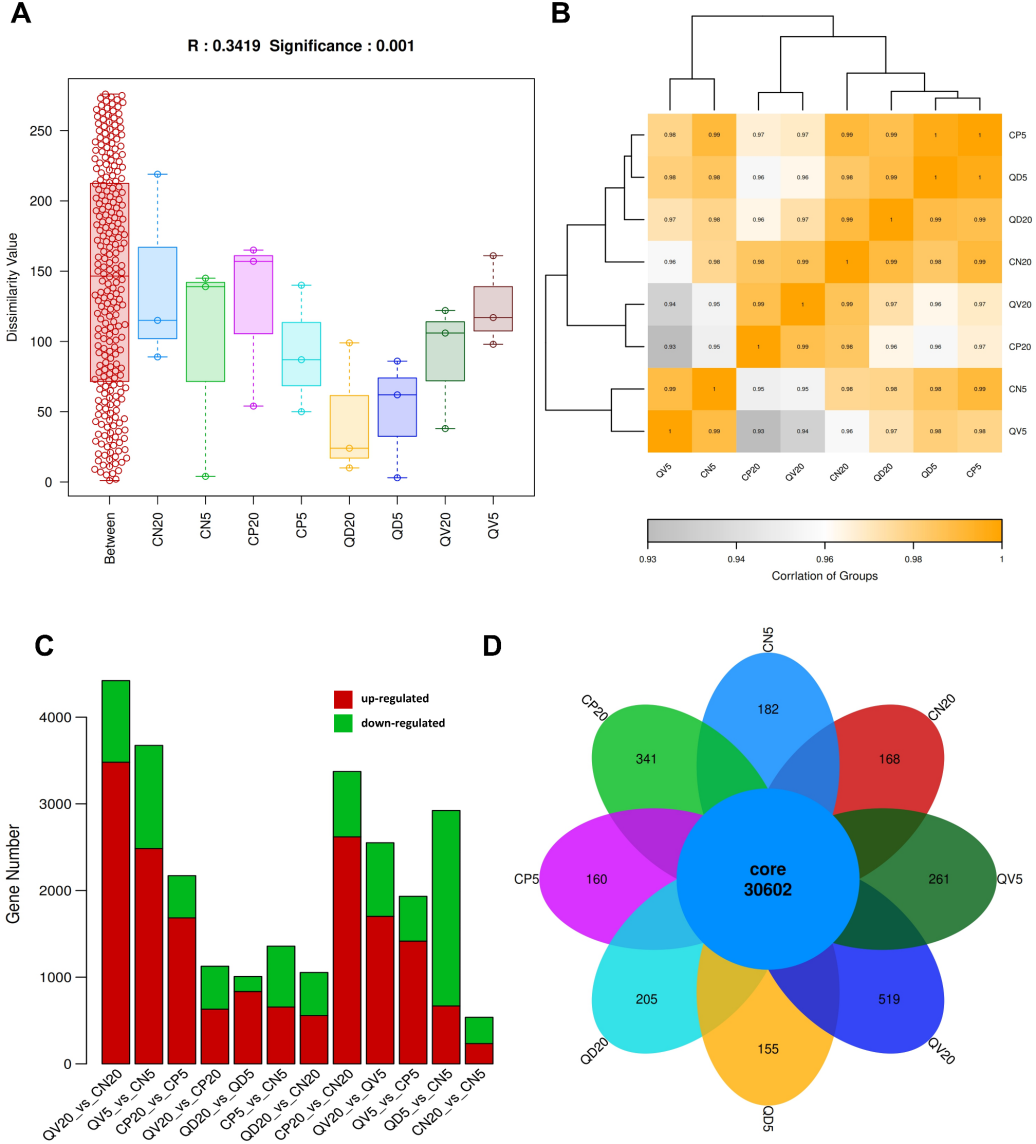
Comparative transcriptome and statistical analysis results among different comparison groups at 5 and 20 dpi. (**A**) Inter- and intragroup analysis of similarity (ANOSIM), (**B**) heatmap depicting TPM distances between eight comparative groups, (**C**) comparative differential gene regulation among various groups in response to different treatments, and (**D**) the number of shared and unique genes expressed are shown in a Venn diagram

To determine the transcriptional responses induced by CLCuMuV infection and CQDs treatment and to elucidate the molecular mechanism/s governing the antiviral activities of the CQDs, we performed comprehensive transcriptome profiling of healthy, CLCuMuV-infected and CQDs-treated plants at 5 and 20 dpi. A total of 24 cDNA libraries were subjected to Illumina sequencing, generating a total of 267,503,324, 270,149,788, 271,651,152 and 271,323,490 raw reads from healthy uninfected, CLCuMuV-infected, CQDs-treated and CQDs-treated + CLCuMuV-infected plants, respectively (Table 1). After cleaning and data quality checking, the results revealed that the Q10, Q20 and Q30 percentages for 24 samples ranged from 99.4 to 99.73, 98.22–99.11 and 95.31–97.33%, respectively. The GC contents ranged between 45.0% and 46.65% (Table 1). The results of ANOSIM statistical analysis revealed that the dissimilarity values within and between different groups were significant (*P* = 0.001; *R* = 0.3419) (Fig. 5A). To further analyze the correlation and similarity between different comparative groups, correlation matrix analysis and Bray‒Curtis beta diversity were performed (Fig. 5B, Supplementary Fig. 3A). A comparative analysis of a total of 61,328 DEGs revealed that at 5 dpi, CLCuMuV infection significantly induced the expression of 1358 genes, among which 656 were upregulated and 705 were downregulated. At this time point, the CQDs-treated plants that exhibited viral infection presented a greater number of significant DEGs, at 3674. Among these DEGs, 2484 were upregulated, whereas 1190 were downregulated (Fig. 5C, Supplementary Fig. 3B; Table 2). The number of shared and unique genes is shown via a Venn diagram, which suggested that a total of 30,602 expressed genes were shared among all groups and that the individual groups had specific expression levels ranging between 155 and 519 (Fig. 5D). Furthermore, at 20 dpi, viral infection significantly induced 3373 DEGs, with 2619 upregulated and 754 downregulated. In the presence of CLCuMuV infection, the application of CQDs induced a relatively greater number of significant DEGs. Among the 4422 DEGs, 3480 were upregulated, and 942 were downregulated (Fig. 5C, Supplementary Fig. 3B; Table 2). Taken together, the results demonstrated that, compared with plants infected with CLCuMuV alone, the CQDs-treated plants presented many more DEGs, and the pattern of expression (up-/downregulation) differed among the comparative groups.

**Table 1:** Statistical summary of 24 RNA-seq libraries from healthy and CLCuMuV-infected *N. benthamiana* (with and without CQDs treatment) at 5 and 20 dpi

Sample	Total reads	Total bases	Q10 (%)	Q20 (%)	Q30 (%)	GC (%)
CN1	50,848,674	7,370,368,330	99.63	98.85	96.73	45.38
CN2	43,611,994	6,236,031,581	99.62	98.81	96.57	45.88
CN3	42,838,452	6,425,645,632	99.65	98.89	96.82	46.26
CN4	41,662,058	6,146,838,475	99.63	98.82	96.60	45.63
CN5	41,569,954	6,104,914,445	99.64	98.85	96.71	46.09
CN6	46,972,192	6,939,790,442	99.62	98.77	96.43	46.65
CP1	40,934,270	6,015,251,150	99.66	98.93	96.91	46.03
CP2	42,367,528	6,235,781,882	99.63	98.84	96.68	46.12
CP3	48,991,530	7,240,292,708	99.66	98.90	96.80	46.29
CP4	45,923,526	6,800,508,805	99.58	98.61	95.92	45.37
CP5	45,825,638	6,790,486,625	99.63	98.82	96.60	45.83
CP6	46,107,296	6,786,919,897	99.66	98.93	96.95	45.76
QD1	47,970,218	7,080,970,636	99.51	98.51	95.96	46.08
QD2	51,978,042	7,717,406,429	99.40	98.22	95.31	46.10
QD3	41,618,084	6,129,103,771	99.66	98.94	96.99	45.89
QD4	44,277,000	6,519,858,033	99.64	98.88	96.80	45.91
QD5	37,223,646	5,473,810,780	99.63	98.84	96.70	46.22
QD6	48,584,162	7,158,545,667	99.73	99.11	97.33	45.90
QV1	53,972,294	7,873,140,615	99.63	98.82	96.60	45.27
QV2	50,999,044	7,526,037,729	99.66	98.92	96.86	45.00
QV3	38,658,572	5,713,056,853	99.56	98.61	96.02	45.89
QV4	44,146,602	6,526,931,014	99.59	98.69	96.20	45.39
QV5	45,371,922	6,624,539,159	99.67	98.98	97.13	45.76
QV6	38,175,056	5,621,667,262	99.64	98.86	96.75	45.24

**Table 2:** Comparative analysis of differentially expressed genes among healthy and CLCuMuV-infected *N. benthamiana* (with and without CQDs treatment) at 5 and 20 dpi

Comparative group	Differentially expressed genes of *N. benthamiana*			
	Total DEGs	Significant DEGs	Significantly upregulated	Significantly downregulated
CP5 vs. CN5	61,328	1358	656	702
QV5 vs. CN5		3674	2484	1190
QV5 vs. CP5		1933	1416	517
QD5 vs. CN5		2923	668	2255
CP20 vs. CN20		3373	2619	754
QV20 vs. CN20		4422	3480	942
QV20 vs. CP20		1127	631	495
QD20 vs. CN20		1054	557	497

* CN, CP, QD and QV represent the groups with negative control (healthy/virus-free), positive control (CLCuMuV-infected), CQDs-treated plants, and CLCuMuV-infected + CQDs-treated plants, respectively. Whereas, 5 and 20 denote the days post CLCuMuV inoculation

### Molecular mechanisms underlying CQDs-mediated antiviral responses in *N. benthamiana*

#### Early stages of CLCuMuV infection

At the early stage of CLCuMuV infection, the number of downregulated genes (702) was greater than that of upregulated genes (656), indicating that viral infection suppressed gene expression at 5 dpi (Supplementary Fig. 4A). We next performed GO analysis to analyze the percentage of genes associated with various functional classes, including biological process, cellular component and molecular function. The GO classification revealed that among 16,067 annotated genes, 464 were associated with cellular processes in the biological process category (q value = 1), 551/19,331 were categorized in the cell part category under the cellular component class (q value = 1), and 304/9555 were associated with catalytic activity under the molecular function class (q value = 0.86058) (Supplementary Fig. 4B). The KEGG pathway classification revealed that ~ 3% (65/2203) of the genes were associated with signal transduction under the environmental information processing category (Supplementary Fig. 4C). KEGG enrichment analysis revealed that 9/291 DEGs were significantly enriched in the phenylpropanoid biosynthesis pathway (ko00940) (Supplementary Fig. 4D). Additional analysis revealed that at 5 dpi, CLCuMuV significantly induced the expression of the *Niben261Chr13g0055007* gene, which is an SCP domain-containing defense-related protein (Supplementary Fig. 5). Compared with that in healthy control plants, the expression of this gene in CLCuMuV-infected plants was significantly greater (*P value* = 0.000160), with a log2 FC of 15.28 and a mean TPM of 3.99486 (Supplementary Fig. 4E; Table 3). In contrast, the expression of *Niben261Chr19g0546002* was significantly suppressed upon viral infection, with a log2 FC value of -14.83 (*P value* = 1.20^E − 05^). The annotation analysis revealed that this gene is associated with the spliceosome/MAPK signaling pathway (Supplementary Fig. 4F; Table 3; Supplementary Fig. 6).

**Table 3:** Important CLCuMuV- and CQDs-regulated candidate DEGs in *N. benthamiana* plants at 5 and 20 dpi

Comparative group*	Gene ID	Annotation	Mean TPM (A vs. B)		Direction	log2 FC	*P*-value (adj)
CP5 vs. CN5	Niben261Chr13g0055007	Plant-pathogen interaction/Defense-related protein containing SCP domain	3.99486	0.0001	up	15.2858	0.000160
	Niben261Chr19g0546002	Spliceosome/MAPK signaling pathway	0.0001	2.91734	down	-14.8323	1.20^E − 05^
QV5 vs. CN5	Niben261Chr14g0615001	Phosphatidylethanolamine binding protein	0.76381	0.0001	up	12.8990	8.82^E − 05^
	Niben261Chr08g0186010	Tam3-transposase	0.0001	0.47317	down	-12.2081	0.004435
QV5 vs. CP5	Niben261Chr01g0881007	Endocytosis/Necroptosis	0.46008	0.0001	up	12.1676	0.006363
	Niben261Chr08g0186010	Tam3-transposase	0.0001	0.35832	down	-11.8070	0.027422
CP20 vs. CN20	Niben261Chr13g0060013	Defense-related protein containing SCP domain	2.93693	0.0001	up	14.8420	5.68^E − 08^
	Niben261Chr15g0895007	MADS-box transcription factor	0.0001	3.45023	down	-15.0744	1.38^E − 07^
QV20 vs. CN20	Niben261Chr13g0060013.1	Defense-related protein containing SCP domain/Plant-pathogen interaction	5.93666	0.0001	up	15.8573	2.40^E − 09^
	Niben261Chr08g0384017.1	Copper chaperone	0.0001	0.61333	down	-12.5824	0.000171
QV20 vs. CP20	Niben261Chr12g0148002	ABC transporters/Sphingolipid signaling pathway	5.83761	0.0001	up	15.8330	0.009172
	Niben261Chr06g0506004	Serine/threonine protein kinase	0.0001	0.20437	down	-10.9969	0.023785

* CN, CP and QV represent the groups with negative control (healthy/virus-free), positive control (CLCuMuV-infected) and CLCuMuV-infected + CQDs-treated plants, respectively. Whereas, 5 and 20 denote the days post CLCuMuV inoculation

**Fig. 6:**
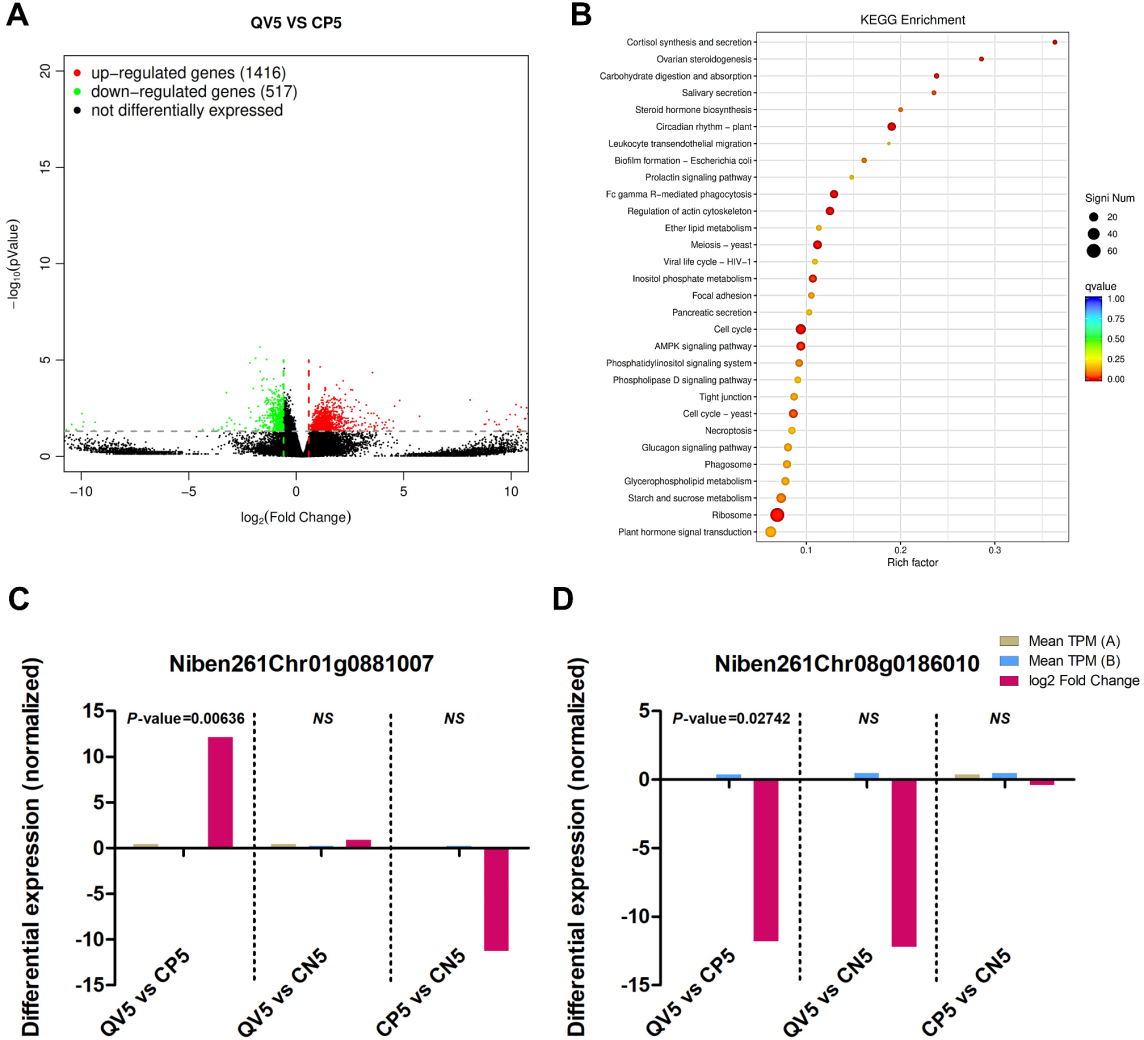
Comparative analysis of different parameters between CQDs-treated + CLCuMuV-infected and virus-infected *N. benthamiana* plants at the early stage (5 dpi) of infection. (**A**) Gene expression is represented by a volcano plot, with each gene signified by a single dot. Red and green dots correspond to significantly up- and downregulated genes, respectively, whereas black dots represent genes that were not differentially expressed in response to viral infection. (**B**) KEGG pathway enrichment analysis representing DEGs associated with specific pathways. The size of the dot represents the number of DEGs significantly enriched in a particular pathway, whereas the color corresponds to the q value being low (0.00) to high (1.00), indicated by red and blue colors, respectively. Differential expression of genes with significantly (**C**) high and (**D**) low expression in response to viral infection

In contrast, CQDs treatment of CLCuMuV-infected plants induced the expression of 1416 genes while suppressing the regulation of 517 genes (Fig. 6A). The results of the GO analysis revealed that most (756/16067) of the genes were associated with cellular processes in the biological process category (q value = 0.4264). However, in the CC class, 901/19,331 genes were linked with the cell part function (q value = 1), and in the MF category, 413/9555 genes (q value = 1) were related to catalytic activity (Supplementary Fig. 7A). Five different classes (cellular processes, environmental information processing, genetic information processing, metabolism and organismal systems) were used to categorize the genes in the KEGG pathway analysis. The results revealed that 52/769, 108/2203, 90/1574, 70/1651 and 47/864 genes were attributed to subgroups including cell growth and death, signal transduction, translation, carbohydrate metabolism and environmental adaptation, respectively (Supplementary Fig. 7B). Finally, KEGG enrichment analysis revealed that 61/489 genes were significantly (*P value* = 0.00006) enriched in the “ribosome” category (ko03010) (Fig. 6B). CQDs application in the presence of CLCuMuV significantly induced the expression of the *Niben261Chr01g0881007* gene, with a mean TPM of 0.46008 and a log2 FC of 12.1676 (*P value* = 0.00636). The annotation analysis revealed that this gene is involved in the endocytosis/necroptosis pathway (Fig. 6C; Supplementary Figs. 8&9). Furthermore, CQDs application significantly suppressed the expression of a Tam3-transposase-related gene (*Niben261Chr08g0186010*), with a mean TPM of 0.35832 and a log2 FC value of -11.8070 (*P value* = 0.02742) (Fig. 6D; Table 3).

### Late-stage CLCuMuV infection

The viral infection of *N. benthamiana* in the absence of CQDs application induced the expression of 26 genes, whereas 754 genes were downregulated (Supplementary Fig. 10A). The percentages of genes in three functional classes (biological process, cellular component and molecular function) were 7.6% (1227/16067), 7.4% (1438/19331) and 7.7% (737/9555), corresponding to cellular process (GO:0009987), cell part (GO:0044464) and catalytic activity (GO:0003824), respectively (Supplementary Fig. 10B). Additional analysis revealed that 78/1064 genes corresponded to the functional subcategory of transport and catabolism; 175/2203 corresponded to signal transduction; 63/1217 were associated with folding, sorting and degradation functions; 120/1651 were involved in carbohydrate metabolism; and 77/864 were linked with environmental adaptation (Supplementary Fig. 10C). Among the DEGs in this group, 66/809 were significantly (*P value* = 0.00008) enriched in the plant hormone signal transduction pathway (ko04075) (Supplementary Fig. 10D). At the late stage of CLCuMuV infection, the expression of the *Niben261Chr13g0060013* gene was significantly (*P value* = 5.6^E − 08^) upregulated, with a mean TPM = 2.93693 and log2 FC = 14.8420 (Supplementary Fig. 10E). In contrast, the expression of a MADS-box transcription factor-related gene (*Niben261Chr15g0895007*) was significantly (*P value* = 1.38E^− 07^) downregulated, with an average TPM value of 3.45023 and log2 FC=-15.0744 (Supplementary Fig. 10F; Table 3).

**Fig. 7:**
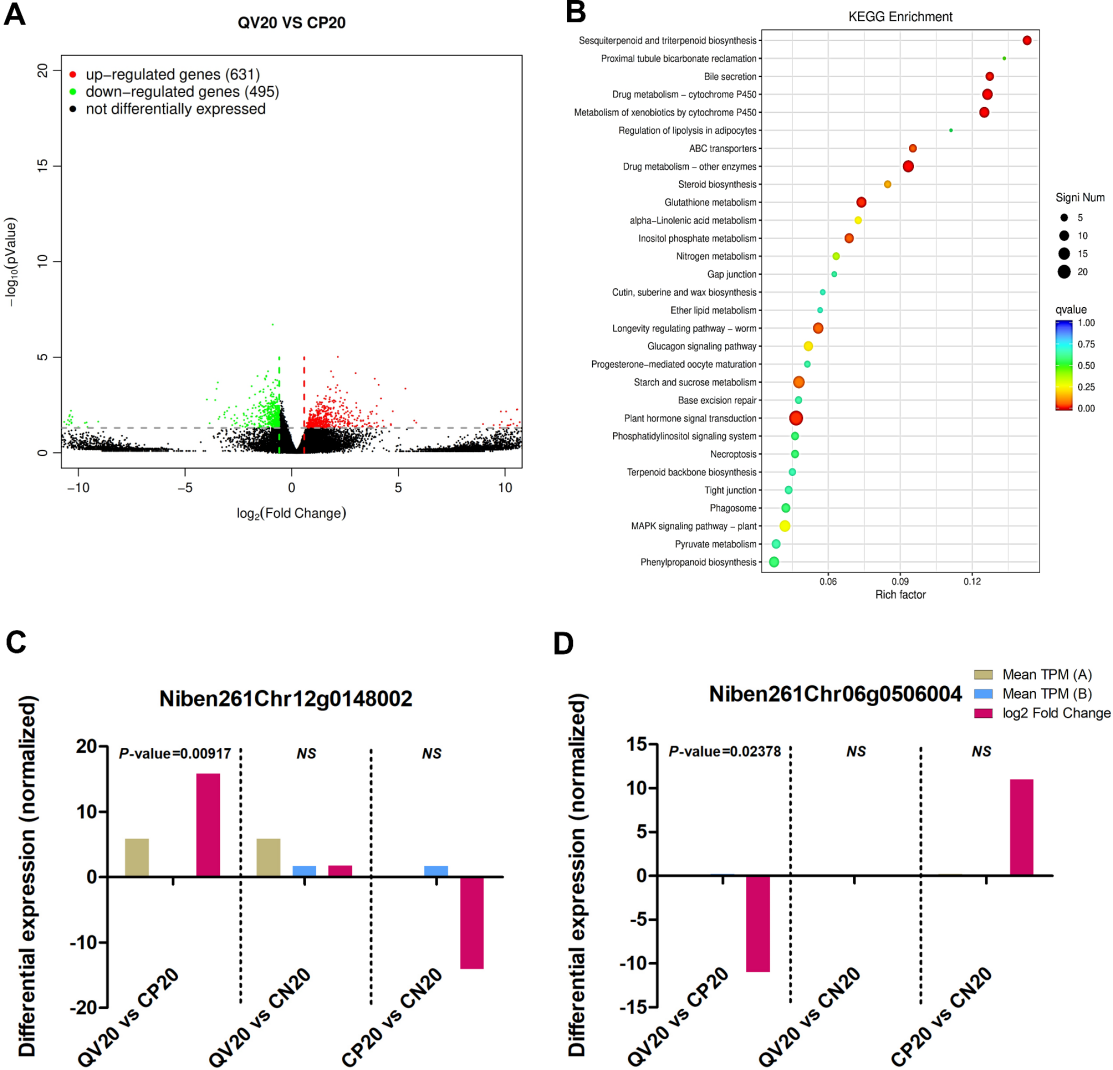
Comparative analysis of different parameters between CQDs-treated + CLCuMuV-infected and virus-infected *N. benthamiana* plants at the late stage (20 dpi) of infection. (**A**) Gene expression is represented by a volcano plot, with each gene signified by a single dot. Red and green dots correspond to significantly up- and downregulated genes, respectively, whereas black dots represent genes that were not differentially expressed in response to viral infection. (**B**) KEGG pathway enrichment analysis representing DEGs associated with specific pathways. The dot size represents the number of DEGs significantly enriched in a particular pathway, whereas the color corresponds to the q value being low (0.00) to high (1.00), indicated by red and blue colors, respectively. Differential expression of genes with significantly (**C**) high and (**D**) low expression in response to viral infection

The gene expression pattern among the CLCuMuV-infected and CQDs-treated plants was altered, in which 631 genes were upregulated and 495 were downregulated (Fig. 7A). The results of GO classification revealed that 392/16,067 genes were associated with cellular processes (GO:0009987), 497/19,331 were linked with cell parts (GO:0044464), and 255/9555 were associated with catalytic activity (GO:0003824) functional subgroups (Supplementary Fig. 11A). In this comparative group, 25/1064 genes were annotated in the transport and catabolism subgroup, 67/2203 in the signal transduction subgroup, 25/1217 in the folding, sorting and degradation subgroup, 44/1651 in the carbohydrate metabolism subgroup, and 24/673 in the endocrine system functional subgroup (Supplementary Fig. 11B). The results of the KEGG enrichment analysis revealed that 24/262 genes were significantly (*P value* = 0.00056) enriched in the plant hormone signal transduction pathway (q value = 0.01419) (Fig. 7B). On the other hand, in the CLCuMuV-infected plants that were treated with CQDs, the *Niben261Chr12g0148002* gene was significantly (*P value* = 0.009172) upregulated, with a mean TPM = 5.83761 and log2 FC = 15.8330 (Fig. 7C). This gene is associated with the ABC transporter/sphingolipid signaling pathway (Supplementary Fig. 12). Additionally, the expression of a serine/threonine protein kinase-related gene (*Niben261Chr06g0506004*) was significantly (*P value* = 0.023785) downregulated, with a mean TPM = 0.20437 and log2 FC= -10.9969 (Fig. 7D; Table 3).

### Variable patterns and frequency of CQDs-induced alternate splicing events among different treatments

**Fig. 8:**
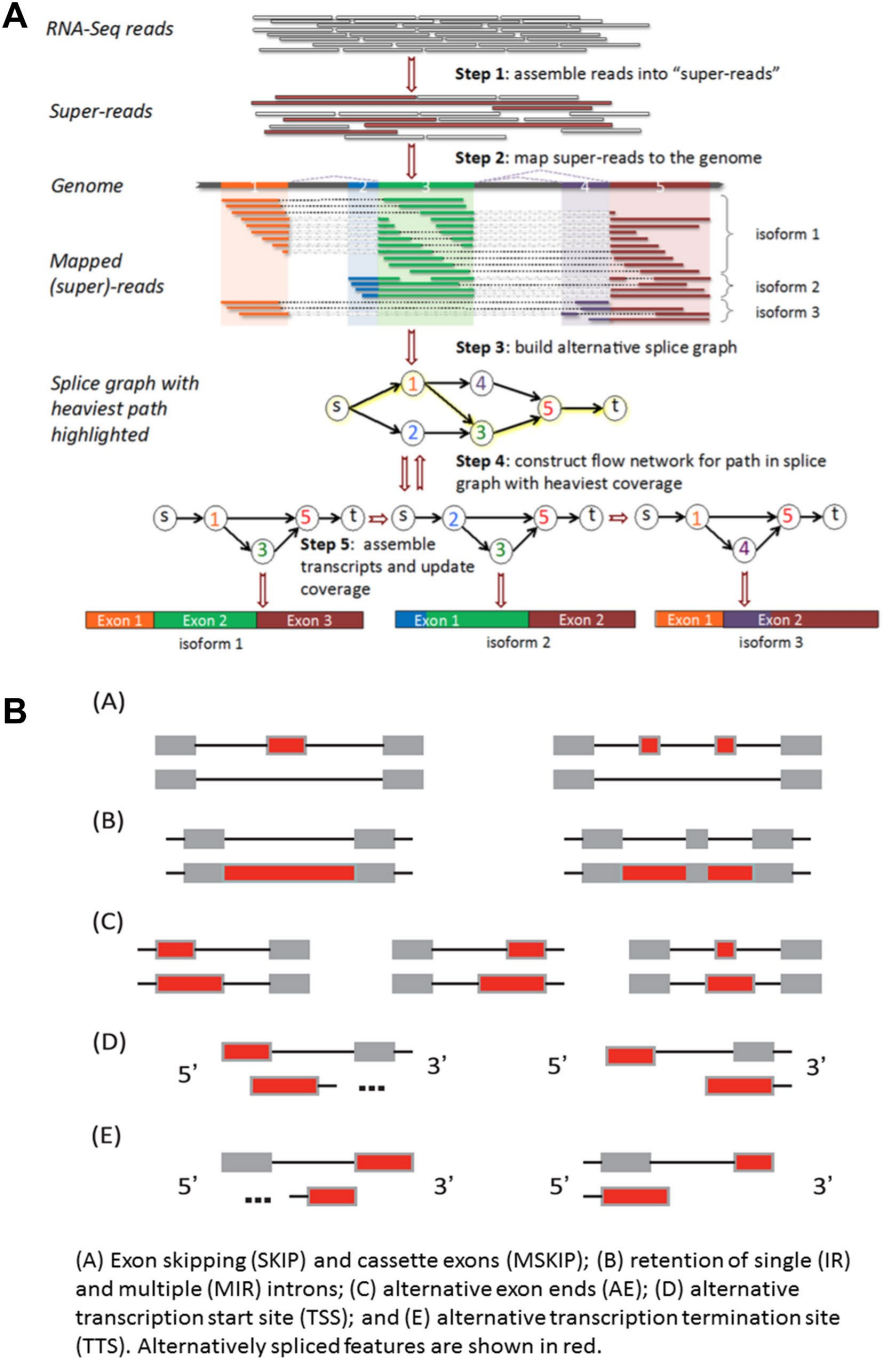
Prediction of new isoforms and alternative splicing events. (**A**) A step-by-step pathway indicating the discovery of new transcripts via RNA-seq data. (**B**) Different types of alternative splicing (AS) events were investigated in this study. These AS types included alternative 5’ first exon (TSS), alternative 3’ last exon (TTS), skipped exon (SKIP), approximate SKIP (XSKIP), multiexon SKIP (MSKIP), approximate MSKIP (XMSKIP), intron retention (IR), approximate IR (XIR), multi-IR (MIR), approximate MIR (XMIR), alternative exon ends (AE) and approximate AE (XAE)

**Fig. 9:**
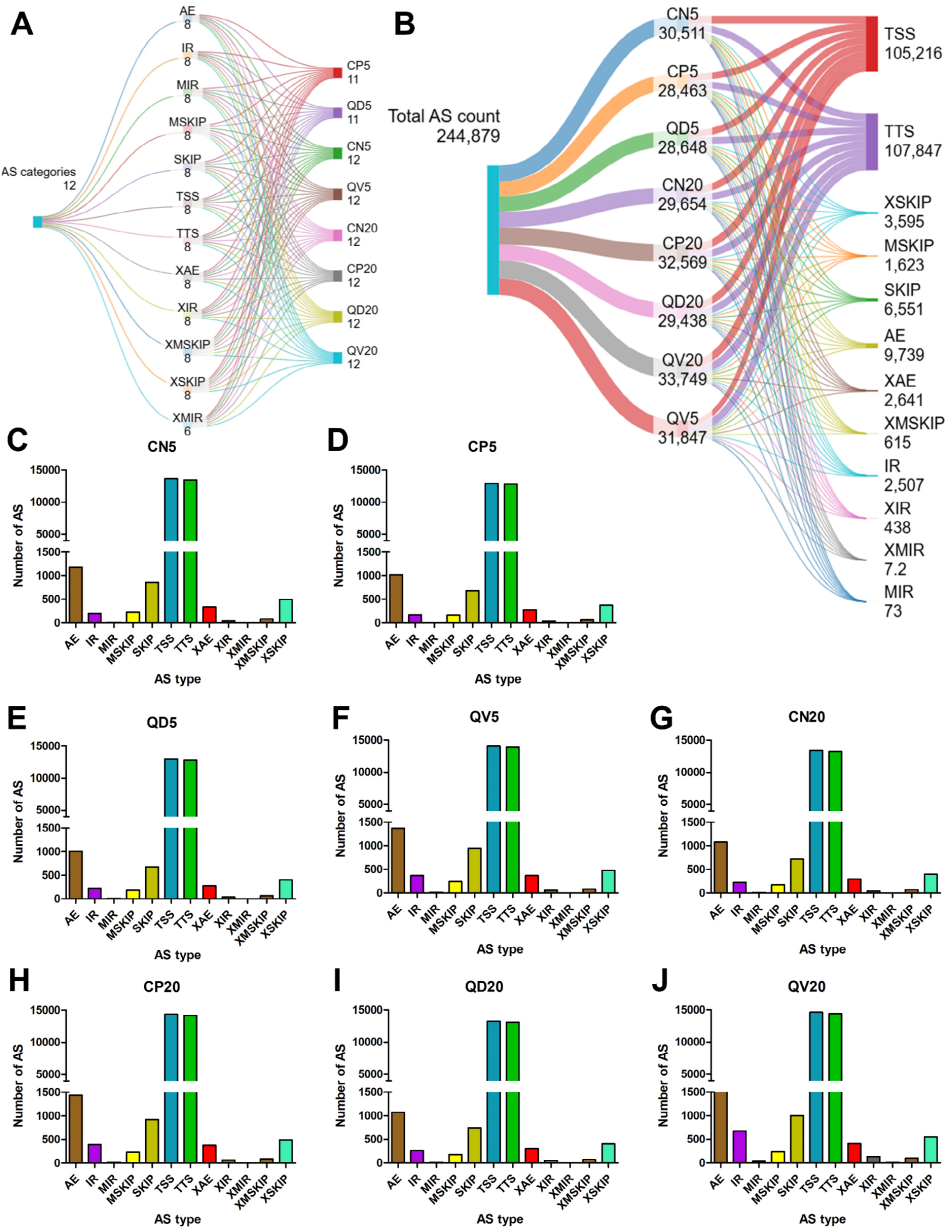
Diversity and frequency of AS events among different comparison groups. (**A**) Types of AS classes detected in healthy and CLCuMuV-infected plants with and without the application of CQDs at 5 and 20 dpi. (**B**) Number of AS events in total and corresponding to specific categories. (**C**-**J**) Comparison of detection frequencies associated with each AS category among different treatments. CN, CP, QD and QV represent the groups with negative control (healthy/virus-free), positive control (CLCuMuV-infected) CQDs-treated and CLCuMuV-infected + CQDs-treated plants, respectively. In contrast, 5 and 20 denote the days post-CLCuMuV inoculation. The vertical axis corresponds to the number of AS events, whereas the horizontal axis denotes the AS category

Given that alternative splicing (AS) is a unique regulatory mechanism that changes host transcript and protein diversity and contributes to various stress-related responses, including plant immunity [38] and pro- or antiviral functions, we sought to determine how CQDs application alters transcript diversity and changes the AS patterns of CLCuMuV-infected plants. We first used RNA-seq data to construct “super-reads” and then mapped those super-reads to the genome to identify unique isoforms (Fig. 8A). Then, the splice graphs with the heaviest coverage were generated, and the assembled transcripts were updated to identify various types of AS (Fig. 8B). The uniquely identified new isoforms are given in Supplementary Table 4. A total of 12 AS categories (AE, IR, MIR, MSKIP, SKIP, TSS, TTS, XAE, XIR, XMSKP, XSKIP and XMIR) were identified in eight comparative groups. All the comparative groups had variable numbers of 12 AS categories except for the QD5 group, in which XMIR was not found (Fig. 9A). Among all the comparative groups, we identified a total of 244,879 AS events. Among these groups, the CQDs-treated groups (QV5 and QV20) presented the highest AS counts (31,847 and 33,749, respectively) in the presence of viral infection at 5 and 20 dpi (Fig. 9B). This clearly demonstrated that, compared with plants infected with CLCuMuV alone, the CQDs-treated plants presented the highest AS diversity. With respect to the AS type, we observed that TSS (105,216) and TTS (107,847) were the most frequently detected AS events among all the comparative groups, whereas MIR and XMIR were the least frequently detected AS types (Fig. 9B-J). Specifically, the TSS count in the CLCuMuV-infected plants (CP5) was 12,903 at 5 dpi, whereas it was 14,046 in the CQDs-treated, virus-infected plants (QV5) (Fig. 9D&F). Additionally, the TTS counts were 12,785 and 13,872 among virus-infected plants only (CP5) and those with CQDs treatment (QV5), respectively (Fig. 9D&F). Similarly, a pattern of AS count was observed at 20 dpi, where the TSSs were 14,376 and 14,637 for the CP20 and QV20 groups, respectively. However, the TTS counts for the CP20 and QV20 comparison groups were 14,175 and 14,402, respectively (Fig. 9H&J). Taken together, these results demonstrate that CQDs treatment of CLCuMuV-infected plants significantly enhances AS diversity at both the early and late stages of infection.

### Validation of candidate gene expression via RT‒qPCR

**Fig. 10:**
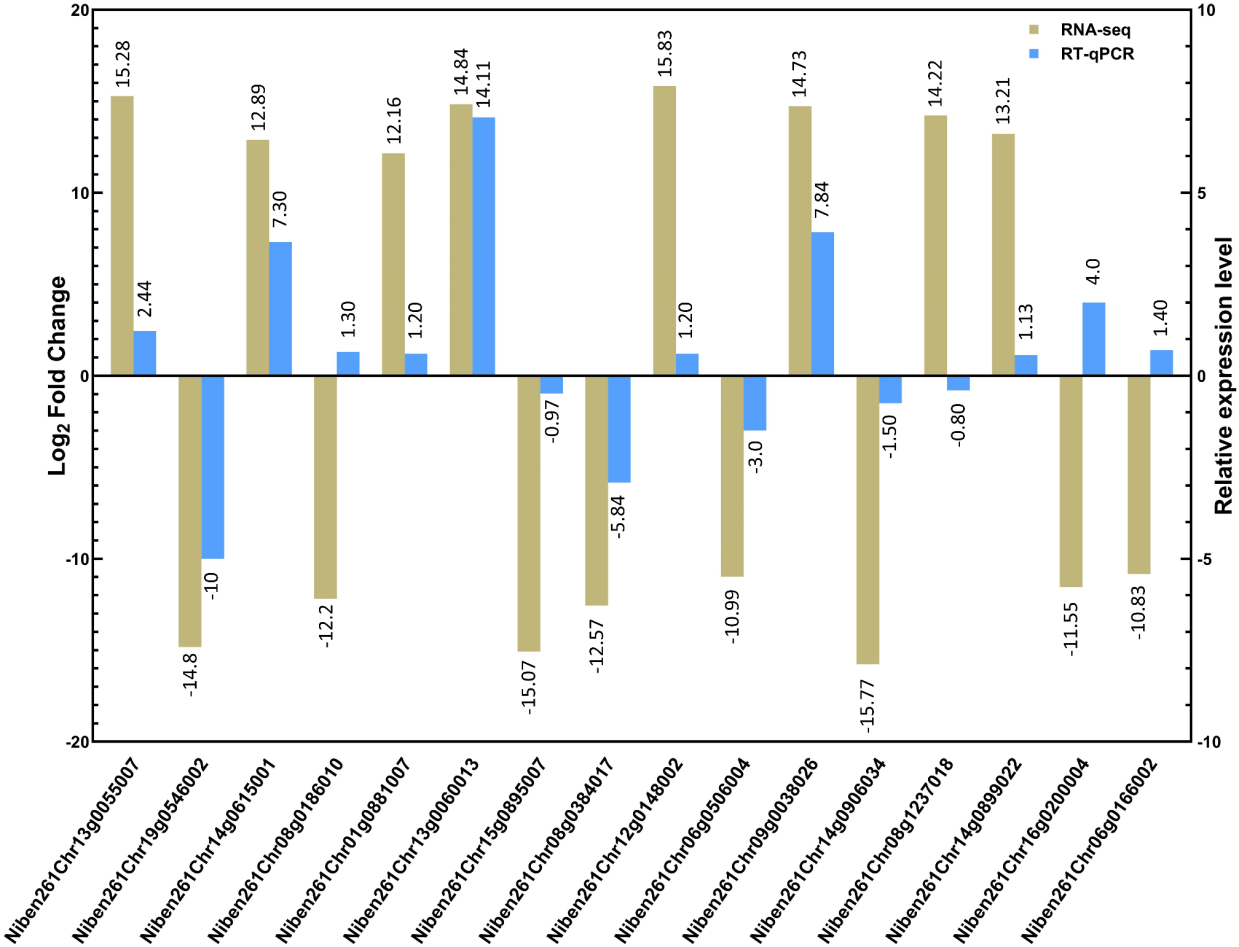
RT‒qPCR validation and comparison of gene expression data obtained from RNA-seq analysis. A total of 16 genes from different comparative groups were selected (details are given in the methods section). Each experiment was replicated with three independent biological replicates, and at least three technical replicates were included per biological replicate (*n* = 9)

To validate the DEG expression data obtained from the RNA-seq data, we compared the mRNA expression profiles of 16 DEGs (Supplementary Table 2). Three different criteria (described in the methods section) were used to select the candidate genes for RT‒qPCR analysis. Among these genes, 12 genes presented concordant expression, whereas 4 genes presented different expression patterns for both RNA-seq and RT‒qPCR (Fig. 10). Generally, the fold change intensity of mRNA expression obtained via RT‒qPCR was lower than that obtained via RNA‒seq (Fig. 10), which could be attributed to the relatively high sensitivity of the RNA‒seq technique. In summary, the directional expression changes obtained via RNA-seq were validated via RT‒qPCR, indicating the high accuracy and reliability of the DEGs identified in our study.

## Discussion

Plants have been fighting viruses throughout history, which has adversely affected sustainable food production systems, resulting in severe and irreversible socioeconomic impacts [39]. Recently, nanotechnology research involving CQDs has focused on revolutionizing the discovery and development of antiviral drugs that could significantly limit viral infections by augmenting plant immunity and photosystems by targeting core pathways in viral disease induction and causal inference [15, 23]. In general, systemic virus infection affects photosystems by decreasing total chlorophyll (*Chl*) and carotenoid contents and reducing the efficiency of CO_2_ assimilation and *photosystem II* photochemistry [40–42]. In our study, we found that the application of fluorescent CQDs significantly increased the morphophysiological properties of CLCuMuV-infected *N. benthamiana* plants, including the plant biomass (leaf area and fresh weight) and photosynthetic performance, by increasing the chlorophyll (*Chla* and *Chlb*) content and photosynthetic performance (*Fv/Fm*, *QY_max*, *NPQ* and *Rfd*), thereby suppressing the viral titer and development of typical symptoms of CLCuMuV. Given that chloroplast organelles and the photosynthetic machinery play vital roles in complex plant‒virus interactions via the regulation of plant defenses against phytoviruses [43], improved photosynthesis and chlorophyll contents despite the presence of viral infection indicate that CQDs increase antiviral plant immunity by improving photosynthetic components and performance. This finding is supported by the gene expression analysis, which revealed that the relative accumulation of the viral transcripts was significantly lower in the CQDs-treated plants than in the untreated plants at 5 and 20 dpi, indicating a greater relative viral abundance with pronounced symptoms. As Xu et al. (2023) reported, CQDs can potentially interact with viral proteins and dynamically suppress the systemic infection of PVY [23]. Carbon dots (CDs), which possess wide-ranging physiochemical properties, have been previously used to increase photosynthesis kinetics and biomass production in plant photosystems [44–47] and inhibit viral infections [11, 48, 49]. Although the role of CQDs-mediated antiviral defense against plant viruses has been understudied, several studies involving human/animal viruses have reported that CQDs-induced antiviral mechanisms in which these nanoparticles are directly involved in inhibiting virion attachment to host cells, impeding viral replication, and inducing an innate immune response, ultimately suppressing viral infection [19, 21, 50–52].

During plant‒virus interactions, the innate immune responses of plants regulate the expression of immunity-related genes involved in endocytosis/necroptosis [53], Tam3-transposase [54, 55], the ABC transporter/sphingolipid signaling pathway [56, 57], and serine/threonine protein kinase activities [58]. However, plant viruses disrupt these immune pathways to instigate successful infection [59]. Therefore, increasing the exogenous expression of these immune responses in plants to combat devasting viruses is a serious matter of concern. Interestingly, the application of CQDs to *N. benthamiana* at the early (5 dpi) and late (20 dpi) stages of CLCuMuV infection resulted in differential gene transcriptional profiles. At the early stage of CLCuMuV infection, CQDs treatment significantly induced the expression of *Niben261Chr01g0881007*, which is involved in the endocytosis/necroptosis pathway, whereas the expression of a Tam3-transposase-related gene (*Niben261Chr08g0186010*) was significantly suppressed. At a later stage, the CQDs regulated the upregulation of the *Niben261Chr12g0148002* gene, which is involved in the ABC transporter/sphingolipid signaling pathway, significantly suppressing the systemic infection of CLCuMuV. These findings are in line with those of Xue et al. (2022), who reported that CQDs trigger host immune responses by increasing the levels of immunoglobulin G (IgG) and T cells and increasing splenocyte proliferation, which could activate cellular and humoral immune pathways against viral infection [14]. Previously, it was reported that CDs with ricin toxin binding subunit B (RTB) also induce the expression of genes involved in the upregulation of interleukin-6 (IL-6) and tumor necrosis factor-α (TNF-α) in host cells and increase the expression of mRNAs, indicating an enhanced immunomodulatory response [60]. Similarly, synthesized CQDs inhibit the systemic infection of both viruses, RNA (porcine reproductive and respiratory syndrome virus, PRRSV) and DNA (pseudorabies virus, PRV), by activating type I interferon (IFN-a and IFN-b) responses and initiating and intracellular signaling pathways, leading to increased expression of IFN-stimulated gene-mediated immune responses [61, 62]. More recently, engineered CQDs have potentially been used for reactive oxygen species (ROS) generation to suppress coronavirus disease 2019 (COVID-19) [63, 64]. In plants, CQDs, which carry double-stranded RNA (dsRNA), induce RNAi without affecting endogenous microRNAs (miRNAs) and protect plants from systemic infection by PYV [23]. Hence, these findings establish the antiviral potential of CQDs, which can enhance plant immunity and suppress viral infection, leading to the development of a sustainable and highly effective nanomaterial-based antiviral system.

In contrast to plant‒virus interactions, alternative splicing is well known to govern plant antiviral immunity [65]. In addition to biotic factors (including phytoviruses), different NPs also alter the AS patterns, contributing to transcriptomic and proteomic diversity in the host and thus regulating the intricacies of the cellular processes significantly involved in immunity [66–68]. We studied the effects of CQDs NPs on the transcript and AS diversity/patterns of CLCuMuV-infected plants and reported that CQDs treatment of CLCuMuV-infected plants significantly enhanced the patterns and frequency of AS events both at the early and late stages of infection, ultimately augmenting host immunity and suppressing CLCuMuV infection. Although few studies have explored CQDs-mediated alterations in AS patterns and frequency in the presence of viral infection, mechanistic explanations have been published. For example, selenium nanoparticle (SeNP) treatment in rainbow trout upregulated the splicing factor family (SRSF3, SRSF7, SRSF9, U2AF1, and U2AF2) and pre-RNA splicing factors (ACIN1 and PPRF18) and promoted AS. Furthermore, the phosphatidylinositol signaling system and the plaque kinase-PI3K-Akt signaling pathway are activated in the host [67]. Moreover, zinc oxide (ZnO) NPs also cause splice junction (SJ) expression changes, regulating AS events in mice at the early (3 days) and later (3 months) stages of application. These ZnO-mediated SJ expression changes in genes drive inflammation, oxidative stress, and apoptosis, as well as the induction of AS in genes associated with oxidative stress and the immune response [69]. These studies provide emerging evidence suggesting that CQDs-mediated AS patterns are involved in regulating host cellular processes and immune responses against devastating viral pathogens. Notably, from the perspective of CQDs-induced AS in RNA/DNA-infected plants, research is limited, and further studies are imperative to highlight the underlying mechanisms that enable AS-mediated antiviral immunity in plants. Additional research on how CQDs-triggered new isoforms and protein diversity contribute to various cellular/biological processes would open interesting avenues for exploring and developing nanoscale antiviral strategies.

This study presents for the first time the mechanistic involvement of CQDs NPs in enhancing plant morphophysiological properties and in the upregulation of several antiviral mechanisms, eliciting immune responses during the course of plant–virus interactions. These engineered CQDs, which regulate transcriptomic and proteomic diversity via variable altering splicing, are relatively safe and represent nascent antiviral strategies that circumvent the limitations of the antiviral drug delivery system and provide new insights into nanomediated antiviral drug design and application for sustainable disease management. We envision that our nanoscale antiviral control system will advance the widespread use of CQDs in augmenting the host immune response against other pathogens.

## Conclusion

In this study, we characterized fluorescent carbon quantum dots (CQDs) via advanced techniques, demonstrating their uniform morphology, crystalline structure, and elemental composition. The optimized application of fluorescent CQDs effectively suppressed the development of disease symptoms, significantly reduced the viral titer and augmented the morphophysiological characteristics and photosynthetic performance of the CLCuMuV-infected plants. The CQDs-mediated mitigation of begomoviral effects underlies the activation of plant defense signaling pathways. CQDs altered gene expression patterns and diversified alternative splicing events, influencing host responses to viral infection. These results will help researchers plan, develop and launch nanotechnology-based, sustainable and eco-friendly antiviral strategies. Future research could involve the application of CQDs to generate “nanobionic” plants with improved photosynthetic performance, antiviral immunity and environmental resilience.

## Electronic supplementary material

Below is the link to the electronic supplementary material.

Supplementary Material 1

Supplementary Material 2

## Data Availability

The datasets presented in this study can be found in online repositories. The names of the repository/repositories and accession number(s) can be found below: National Center for Biotechnology Information (NCBI), BioProject number PRJNA1155227.
