# Supplementary material for: Enhanced antiviral defense against begomoviral infection in Nicotiana benthamiana through strategic utilization of fluorescent carbon quantum dots to activate plant immunity
Source: J Nanobiotechnology. 2024 Nov 14;22:707. doi: 10.1186/s12951-024-02994-4 (PMC11562592; doi:10.1186/s12951-024-02994-4)
Supplement: Supplementary file 2 — Supplementary Material 2 [file 12951_2024_2994_MOESM2_ESM.docx]

**
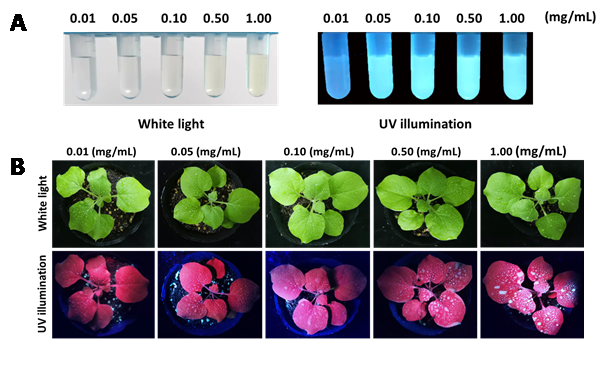
**

**Supplementary Figure 1:** (A) Optical properties of various concentrations of CQDs dissolved in ddH_2_O and (B) appearance under normal white light and UV illumination after foliar spray on *N. benthamiana* plants.


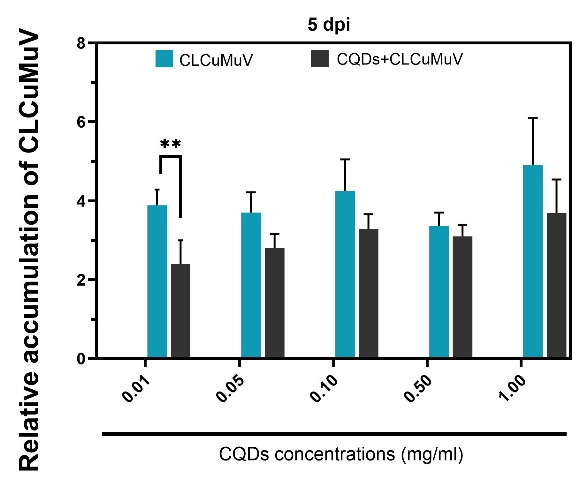

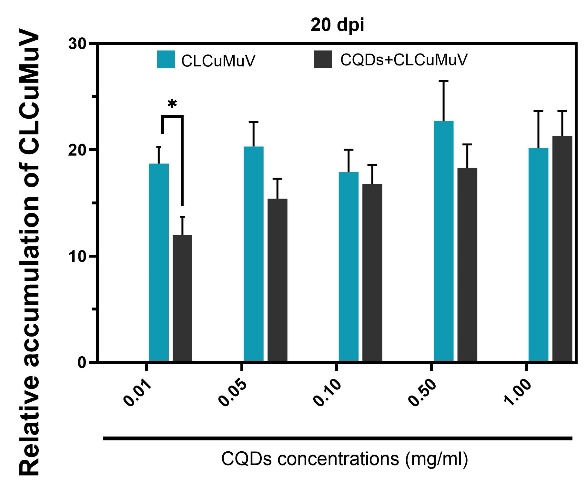


**Supplementary Figure 2:** Relative quantification of CLCuMuV transcripts in virus-infected *N. benthamiana* at 5 and 20 dpi in response to different concentrations of CQDs.


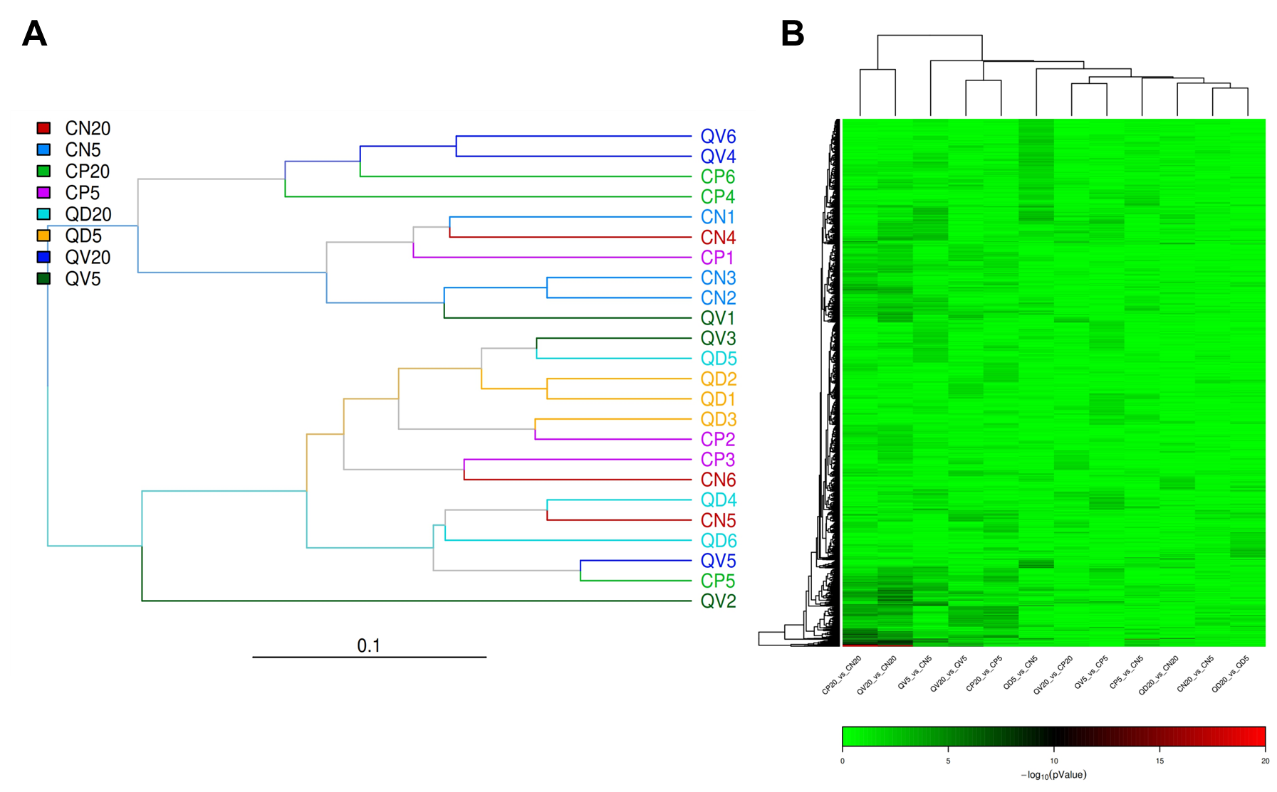


**Supplementary Figure 3:** (A) Hierarchical cluster tree analysis via the Bray‒Curtis statistical algorithm, and (B) heatmap of DEGs corresponding to different groups, including healthy, virus-treated and CQDS-treated plants with and without CLCuMuV infection. The colored scale represents low and high -log_10_(p values) ranging between 0 (green) and 20 (red).


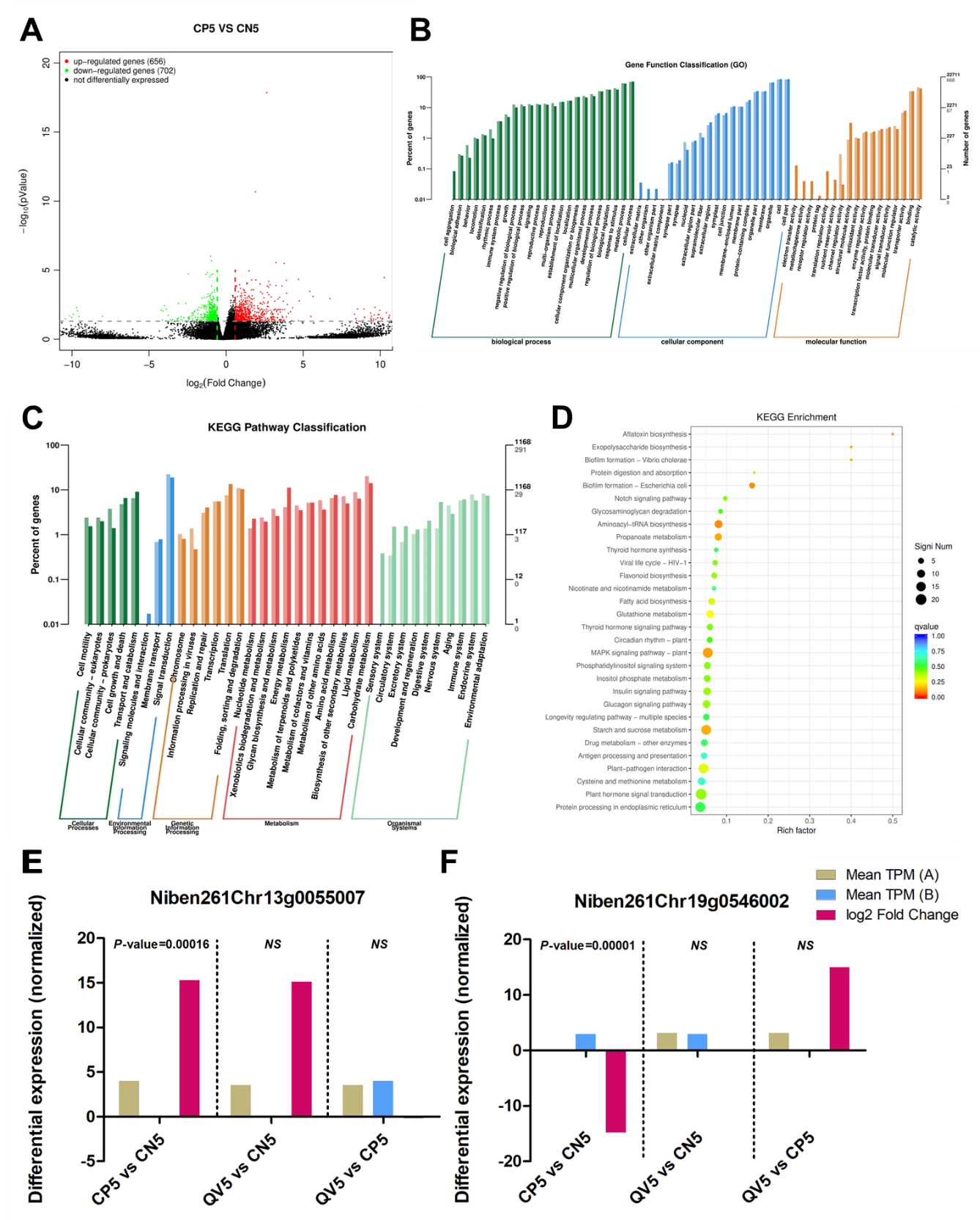


**Supplementary Figure 4:** Comparative analysis of different parameters between CLCuMuV-infected and healthy *N. benthamiana* plants at the early stage (5 dpi) of infection. (A) Gene expression is represented by a volcano plot, with each gene signified by a single dot. Red and green dots correspond to significantly up- and downregulated genes, respectively, whereas black dots represent genes that were not differentially expressed in response to viral infection. (B) Categorization of DEGs into three functional classes via gene ontology (GO) analysis. (C) Kyoto Encyclopedia of Genes and Genomes pathway classification to categorize DEGs into five functional classes. (D) KEGG pathway enrichment analysis representing DEGs associated with specific pathways. The size of the dot represents the number of DEGs significantly enriched in a particular pathway, whereas the color corresponds to the q value being low (0.00) to high (1.00), indicated by red and blue colors, respectively. Differential expression of genes with significantly (E) high and (F) low expression in response to viral infection.


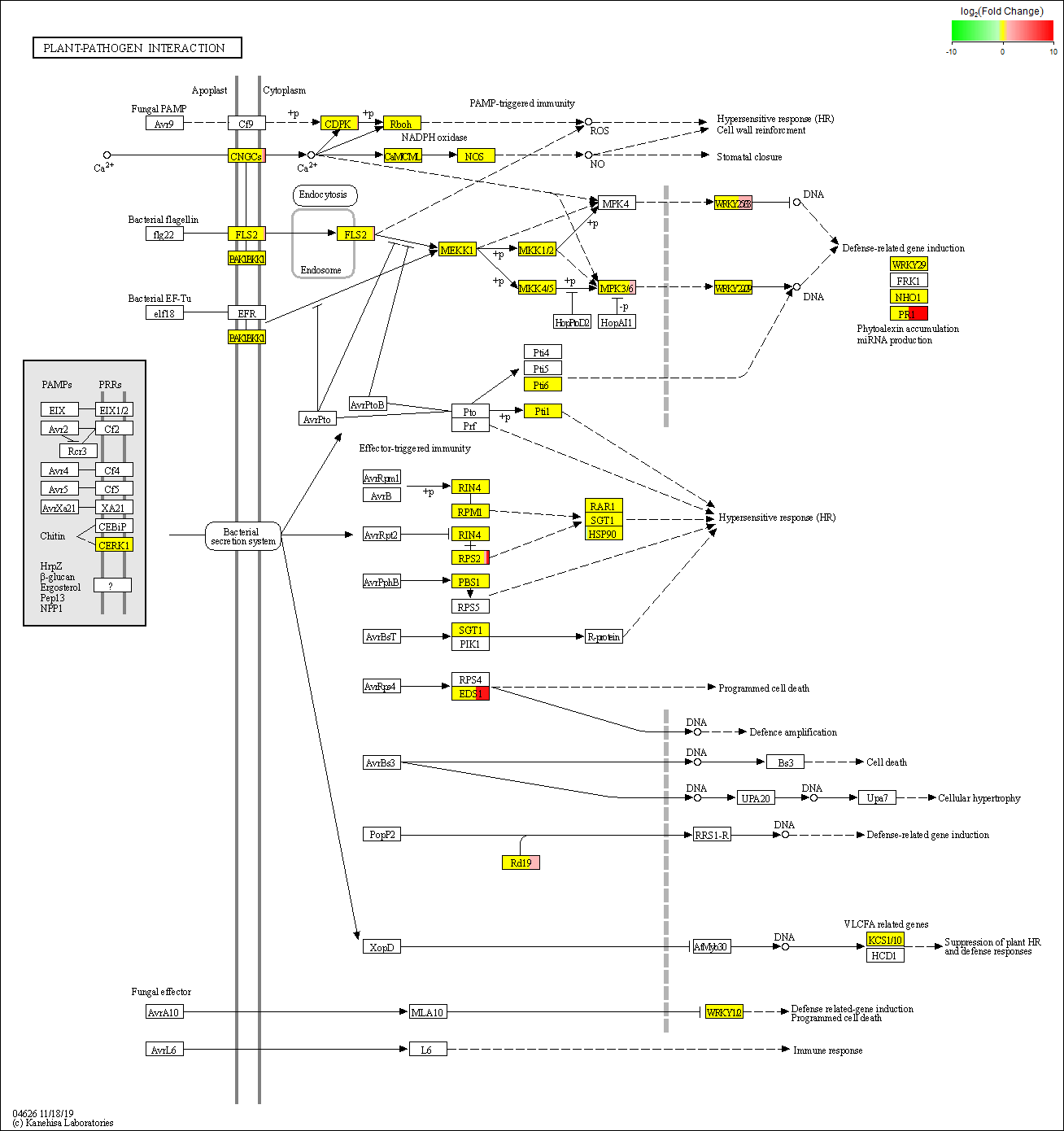


**Supplementary Figure 5:** Plant-pathogen interaction pathway map associated with higher expression of *Niben261Chr13g0055007* in the CP5 VS CN5 comparative group.


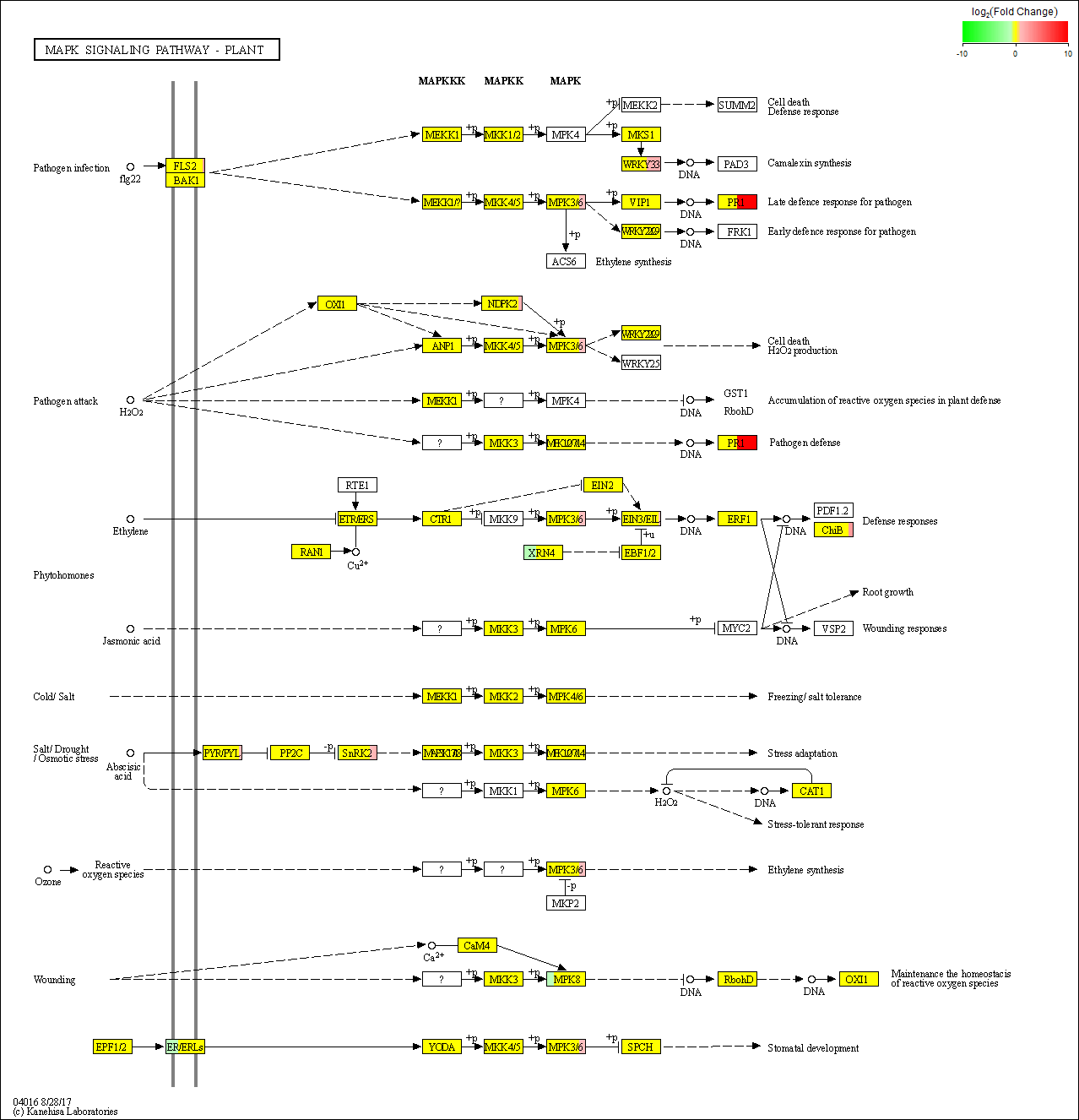


**Supplementary Figure 6:** MAPK-signalling pathway map associated with higher expression of *Niben261Chr19g0546002* in the CP5 VS CN5 comparative group.


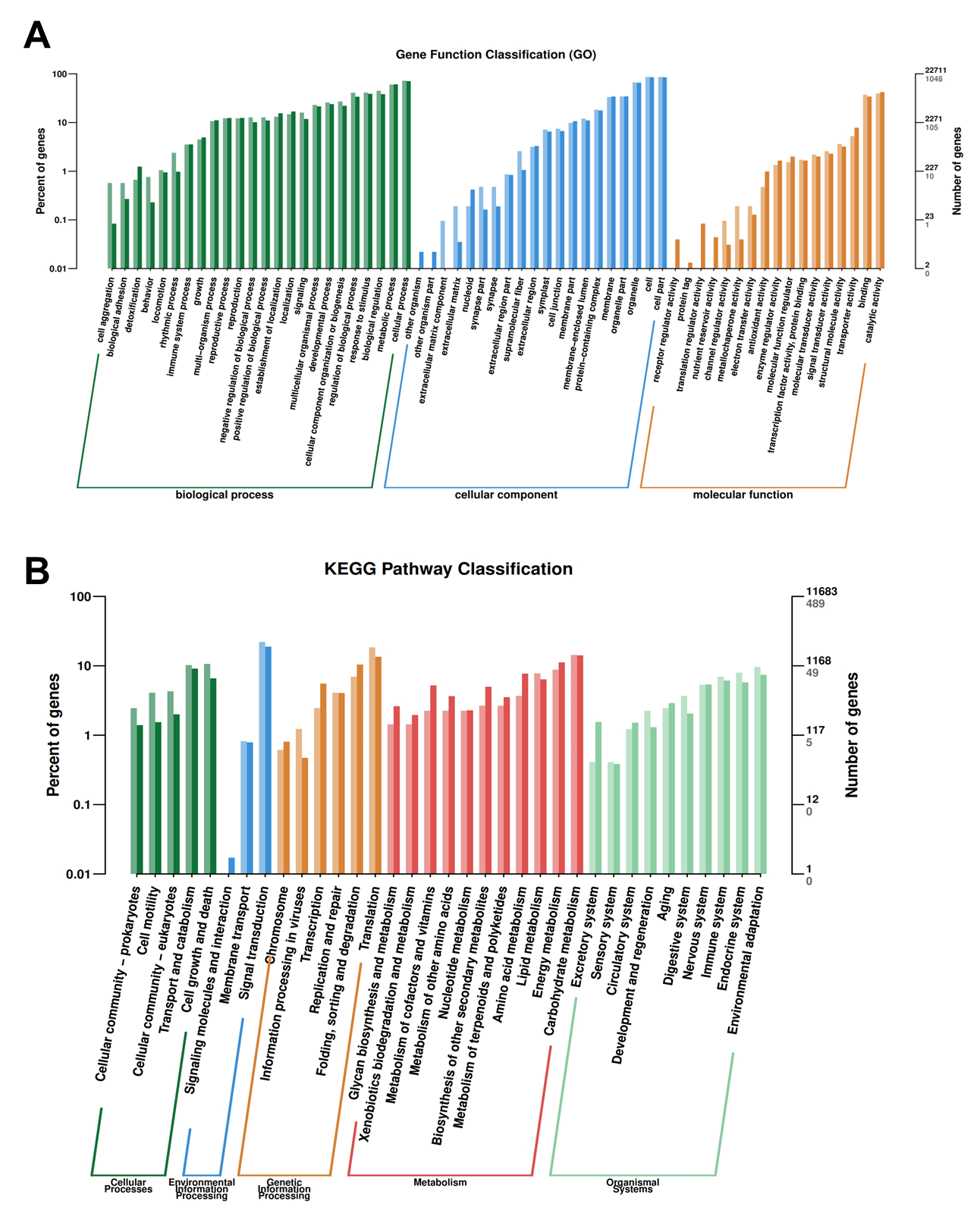


**Supplementary Figure 7:** Gene ontology and KEGG pathway classification of DEGs. (A) Categorization of DEGs into three functional classes via gene ontology (GO) analysis. (B) Kyoto Encyclopedia of Genes and Genomes pathway classification to categorize DEGs into five functional classes.


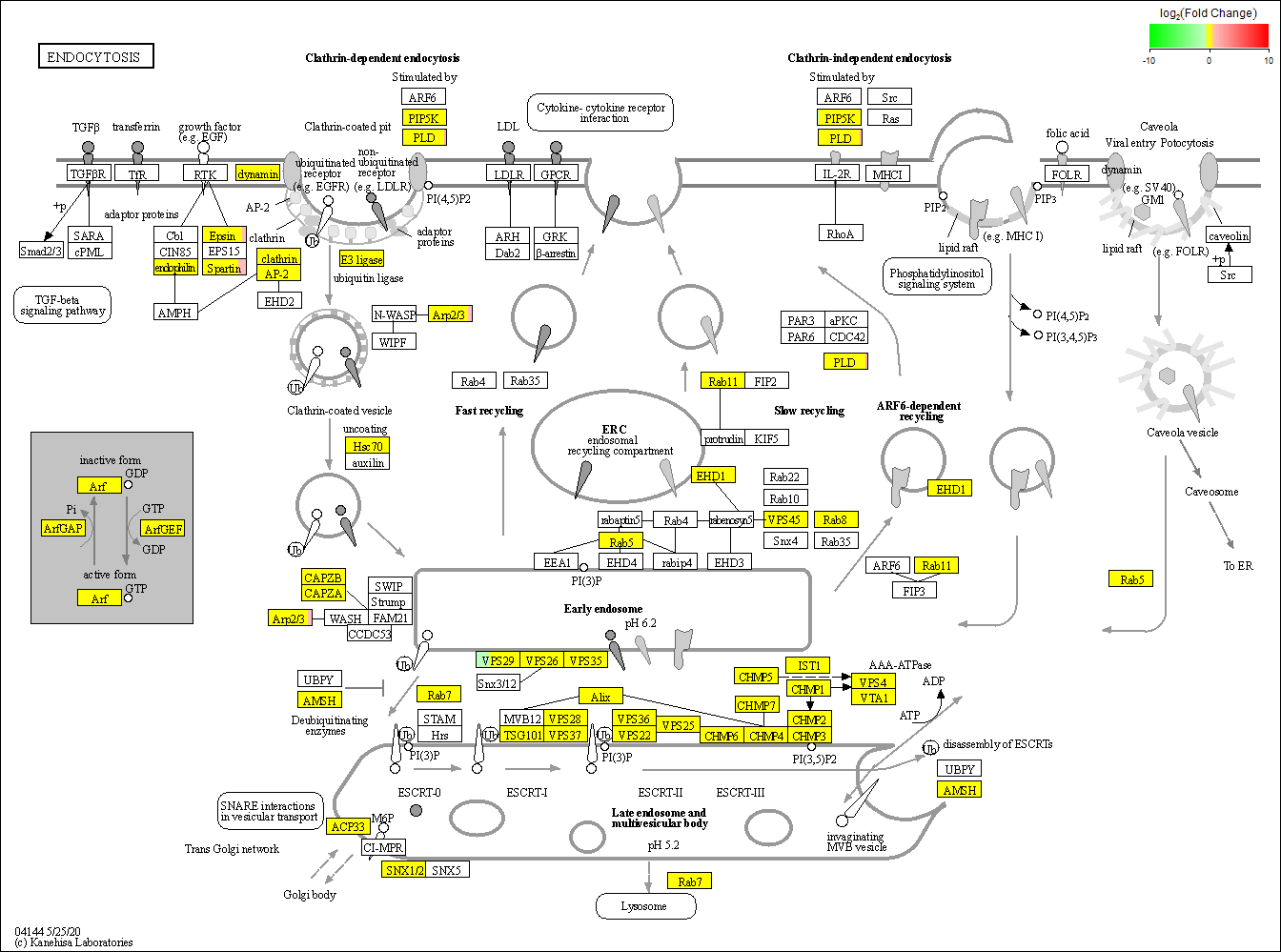


**Supplementary Figure 8:** Endocytosis pathway map associated with higher expression of *Niben261Chr01g0881007* in the QV5 VS CP5 comparative group.


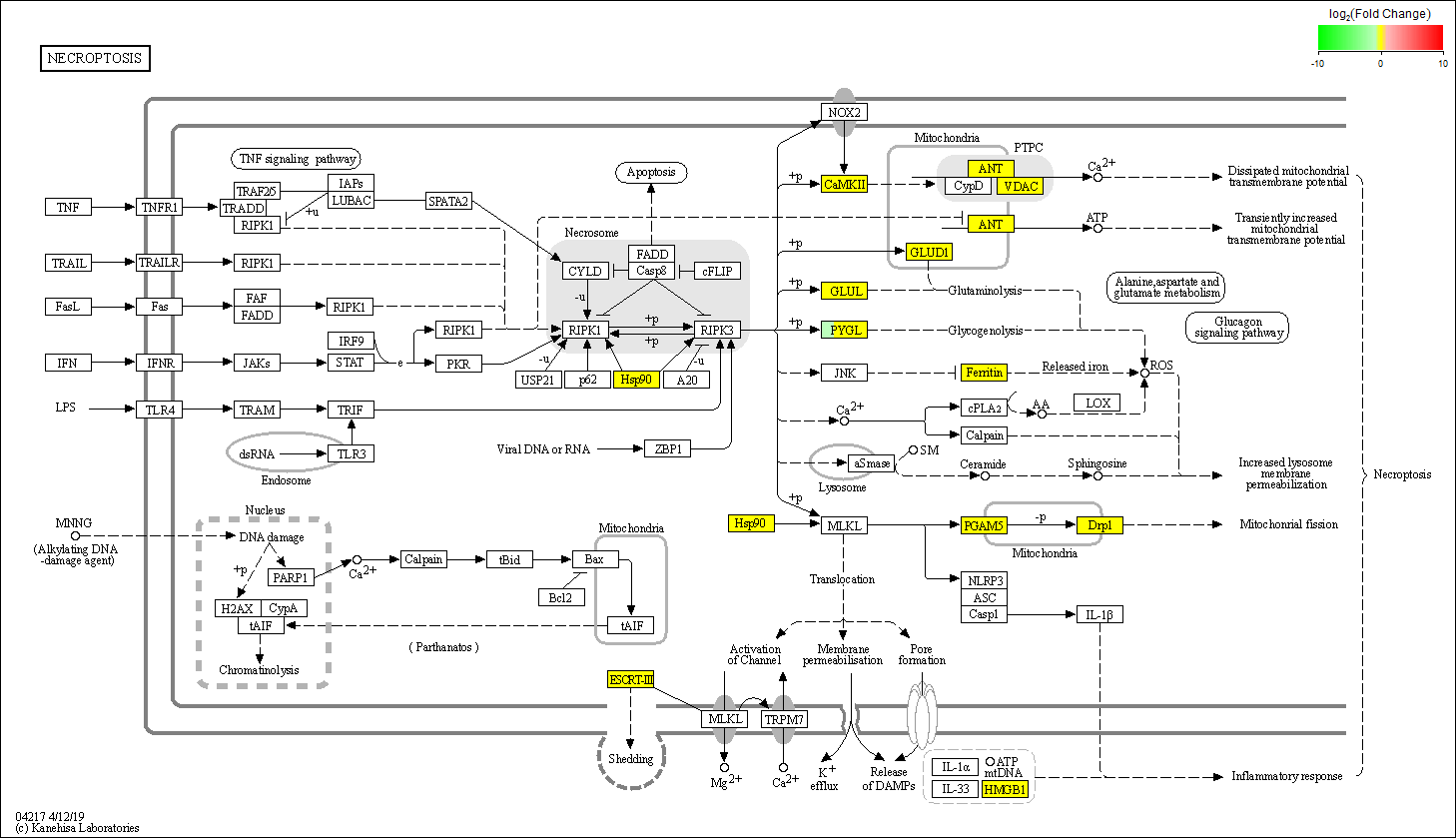


**Supplementary Figure 9:** Necroptosis pathway map associated with higher expression of *Niben261Chr01g0881007* in the QV5 VS CP5 comparative group.


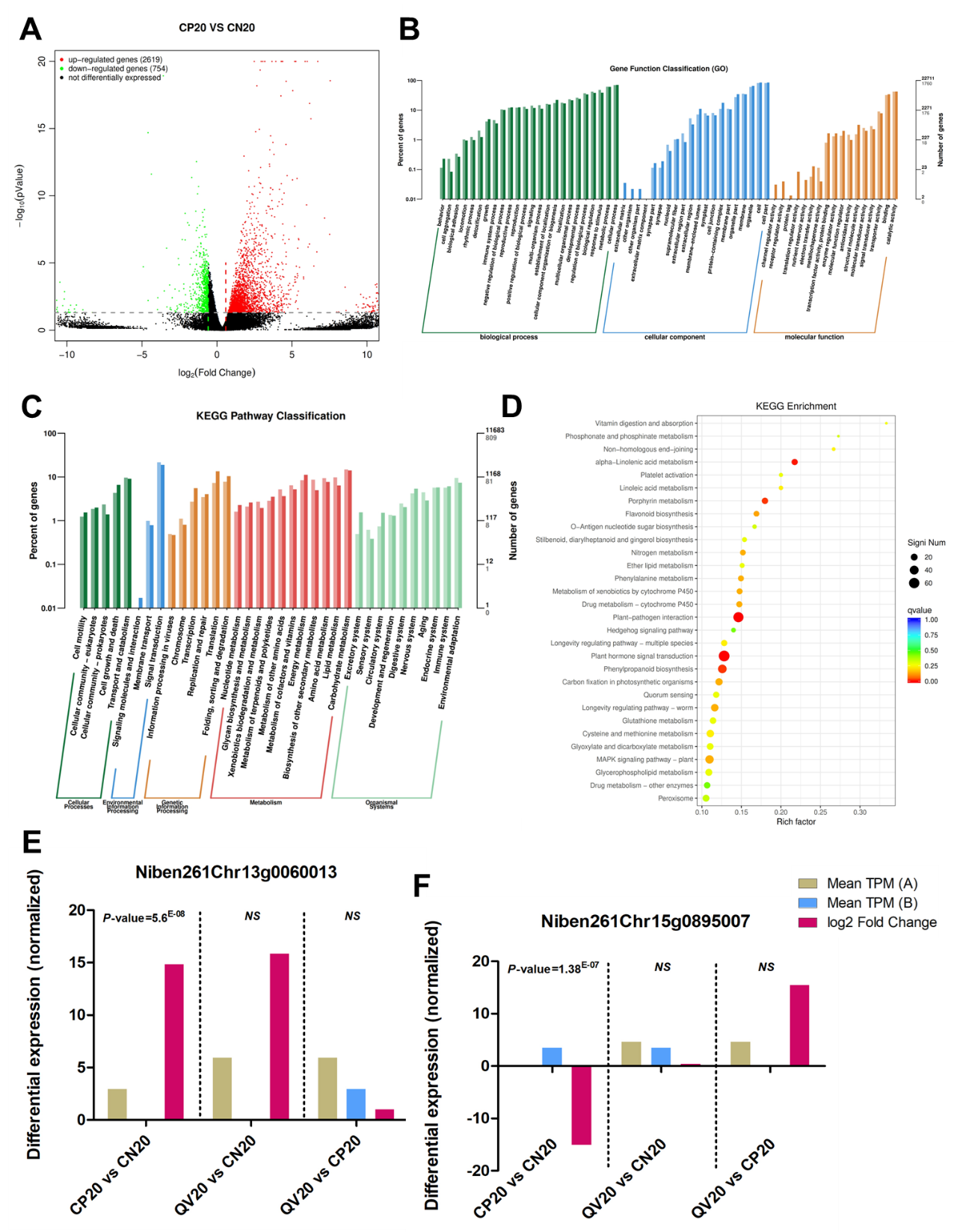


**Supplementary Figure 10:** Comparative analysis of different parameters between CLCuMuV-infected and healthy *N. benthamiana* plants at the late stage of infection (20 dpi). (A) Gene expression is represented by a volcano plot, with each gene signified by a single dot. Red and green dots correspond to significantly up- and downregulated genes, respectively, whereas black dots represent genes that were not differentially expressed in response to viral infection. (B) Categorization of DEGs into three functional classes via gene ontology (GO) analysis. (C) Kyoto Encyclopedia of Genes and Genomes pathway classification to categorize DEGs into five functional classes. (D) KEGG pathway enrichment analysis representing DEGs associated with specific pathways. The dot size represents the number of DEGs significantly enriched in a particular pathway, whereas the color corresponds to the q value being low (0.00) to high (1.00), indicated by red and blue colors, respectively. Differential expression of genes with significantly (E) high and (F) low expression in response to viral infection.


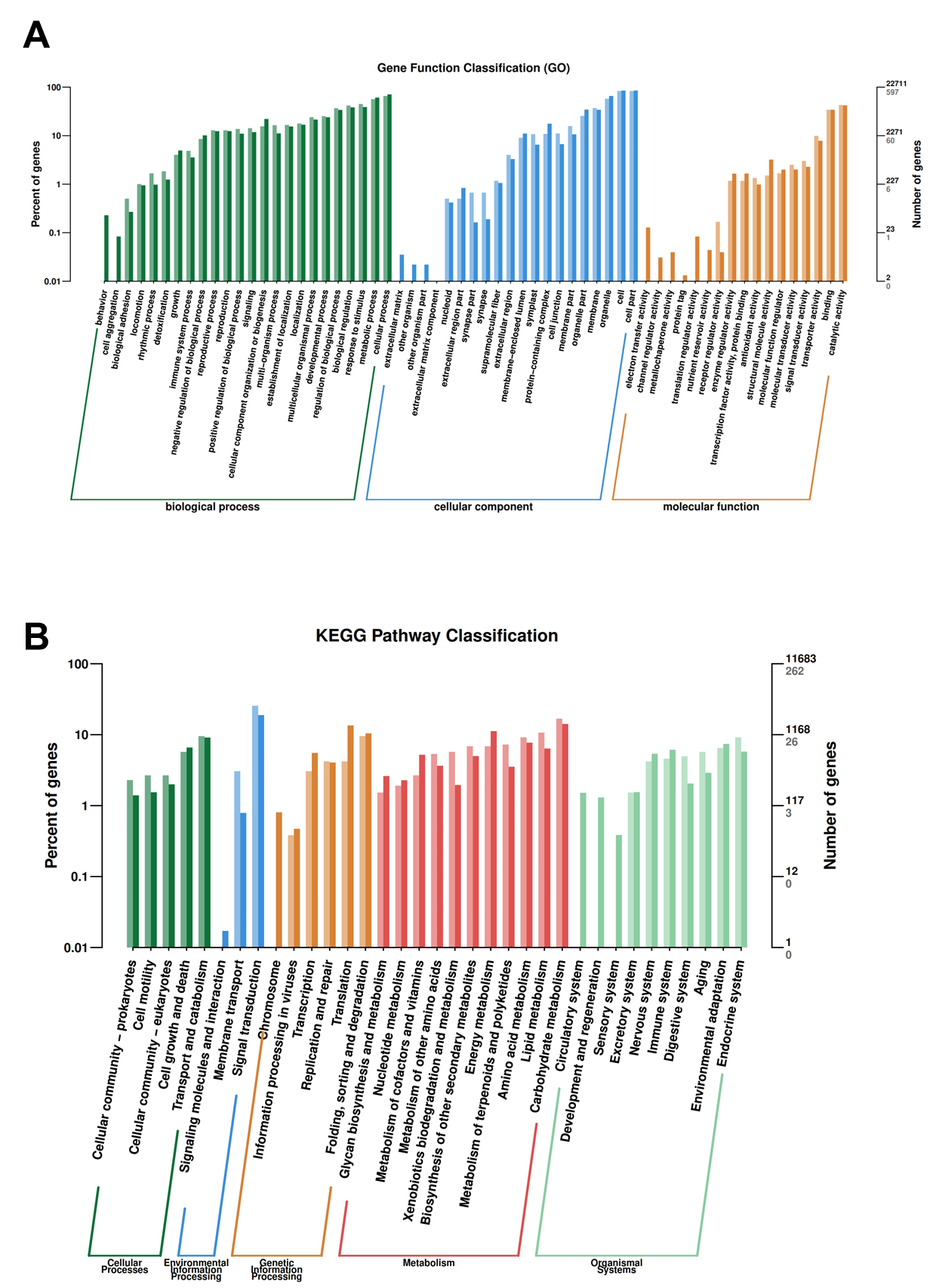


**Supplementary Figure 11:** (A) Categorization of DEGs into three functional classes via gene ontology (GO) analysis. (B) Kyoto Encyclopedia of Genes and Genomes pathway classification to categorize DEGs into five functional classes.
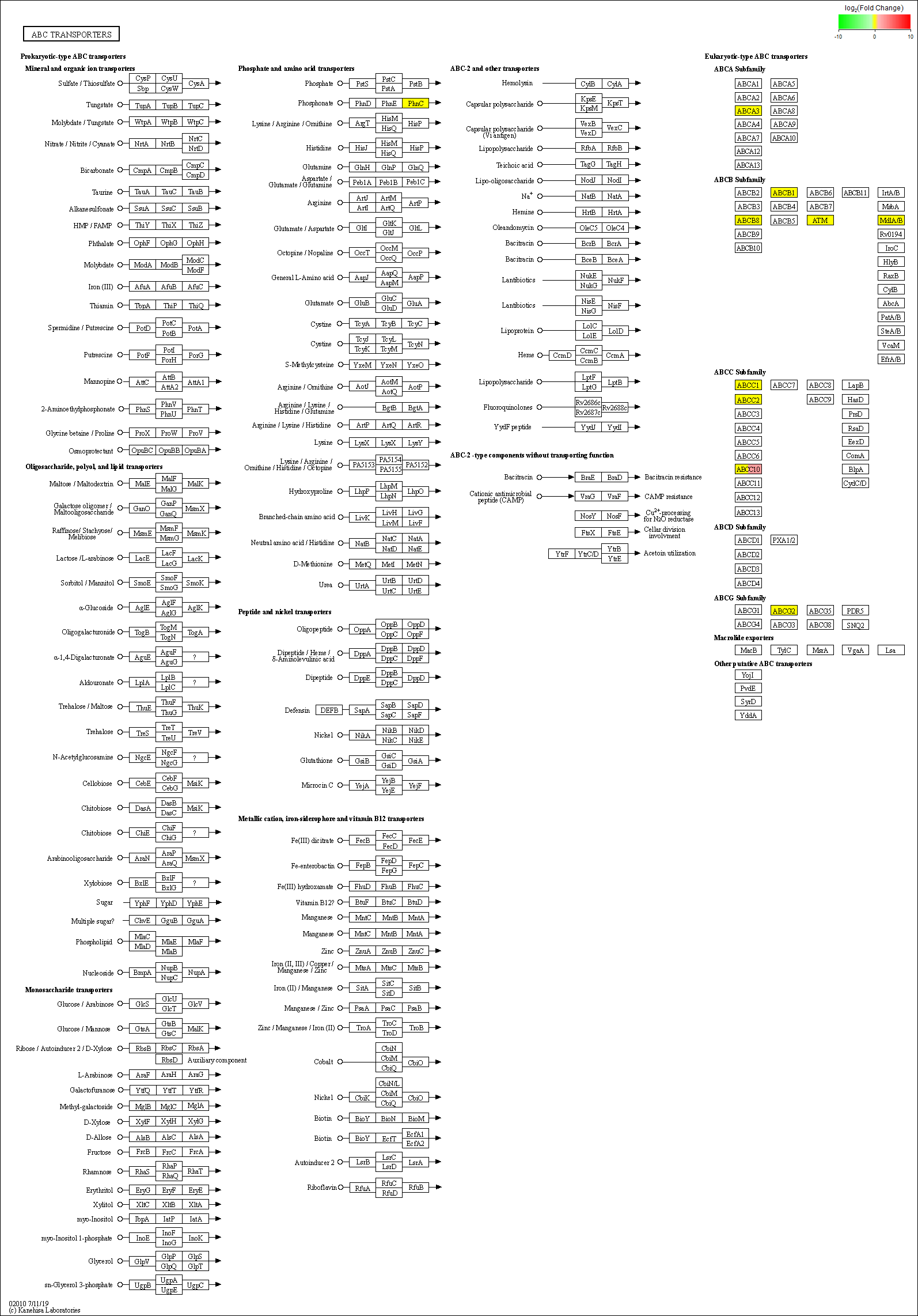


**Supplementary Figure 12:** ABC transporters pathway map associated with higher expression of *Niben261Chr12g0148002* in the QV20 VS CP20 comparative group.

**Supplementary Table 1:** Details of primers used for PCR-based detection of CLCuMuV.

| Primer | Sequence (5'-3') | Product size (bp) | Purpose |
| --- | --- | --- | --- |
| CLCuMuV-CL-F | CAGGAAGCAGGAAAATACGAGA | 831 | CLCuMuV detection |
| CLCuMuV-CL-R | TGGCAGTCCAACACAAAATACG |  |  |
| CLCuMuB-beta-F | AAGTCGAATGGAACGTGAATGT | 837 | CLCuMuV detection |
| CLCuMuB-beta-R | GGAGACCAAAAGAGGAGAGAGA |  |  |

**Supplementary Table 2:** List of candidate genes used for RT-qPCR analysis.

| **No.** | **Gene** | **Annotation** | **Comparative group** |
| --- | --- | --- | --- |
| 1 | *Niben261Chr13g0055007* | K13449-ko04016 MAPK signaling pathway - plant;ko04075 Plant hormone signal transduction;ko04626 Plant-pathogen interaction | CP5 vs CN5 |
| 2 | *Niben261Chr19g0546002* | K03283-ko03040 Spliceosome;ko04141 Protein processing in endoplasmic reticulum;ko04010 MAPK signaling pathway;ko04144 Endocytosis;ko04612 Antigen processing and presentation;ko04915 Estrogen signaling pathway;ko04213 Longevity regulating pathway - multiple species;ko05162 Measles;ko05134 Legionellosis;ko05145 Toxoplasmosis;ko05020 Prion disease;ko05417 Lipid and atherosclerosis | CP5 vs CN5 |
| 3 | *Niben261Chr14g0615001* | KOG3346-Phosphatidylethanolamine binding protein[R]; K16223-ko04712 Circadian rhythm - plant | QV5 vs CN5 |
| 4 | *Niben261Chr08g0186010* | KOG1121-Tam3-transposase (Ac family)[L] | QV5 vs CN5 |
| 5 | *Niben261Chr01g0881007* | K12194-ko03250 Viral life cycle - HIV-1;ko04144 Endocytosis;ko04217 Necroptosis; KOG1656-Protein involved in glucose derepression and pre-vacuolar endosome protein sorting[U] | QV5 vs CP5 |
| 6 | *Niben261Chr13g0060013* | K13449-ko04016 MAPK signaling pathway - plant;ko04075 Plant hormone signal transduction;ko04626 Plant-pathogen interaction; KOG3017-Defense-related protein containing SCP domain[S] | CP20 vs CN20 |
| 7 | *Niben261Chr15g0895007* | KOG0014-MADS box transcription factor[K] | CP20 vs CN20 |
| 8 | *Niben261Chr08g0384017* | KOG1603-Copper chaperone[P] | QV20 vs CN20 |
| 9 | *Niben261Chr12g0148002* | K05665-ko02010 ABC transporters;ko04071 Sphingolipid signaling pathway;ko04977 Vitamin digestion and absorption;ko05206 MicroRNAs in cancer;ko01523 Antifolate resistance; KOG0054-Multidrug resistance-associated protein/mitoxantrone resistance protein, ABC superfamily[Q] | QV20 vs CP20 |
| 10 | *Niben261Chr06g0506004* | KOG0580-Serine/threonine protein kinase[D] | QV20 vs CP20 |
| 11 | *Niben261Chr09g0038026* | N/A | CP5 vs CN5 |
| 12 | *Niben261Chr14g0906034* | N/A | CP5 vs CN5 |
| 13 | *Niben261Chr08g1237018* | N/A | QV5 vs CP5 |
| 14 | *Niben261Chr14g0899022* | KOG0231-Junctional membrane complex protein Junctophilin and related MORN repeat proteins[R] | QV20 vs CP20 |
| 15 | *Niben261Chr16g0200004* | N/A | QV20 vs CP20 |
| 16 | *Niben261Chr06g0166002* | N/A | QV20 vs CP20 |

**Supplementary Table 3:** List of primers used for RT-qPCR analysis

| **No.** | **Gene** | **Forward (5-3')** | **Reverse (5-3')** |
| --- | --- | --- | --- |
| 1 | *Niben261Chr13g0055007* | GTGCAGATGTAGGCGTAGAA | CGCCGTATTGACCATGAGAA |
| 2 | *Niben261Chr19g0546002* | CATTGCCAAGATGTACCAAAGC | GGAATTCTGCGAGTGAACCA |
| 3 | *Niben261Chr14g0615001* | CACAGACATCCCAGCAACTA | CGACCCAATTGTCGAAACAC |
| 4 | *Niben261Chr08g0186010* | TCTCTTAGCCATTCCTGTCTCT | GCACACATTAAAGCCTCCAAAG |
| 5 | *Niben261Chr01g0881007* | TGTGGCAGTGATGGATTTATGT | TACAACAAACAACGGCGAAAC |
| 6 | *Niben261Chr13g0060013* | GGTGATGTAGGCGTCGAAC | TTCGCCGTATTGACCATGAG |
| 7 | *Niben261Chr15g0895007* | GAGAGGGAAGGTAGAAATGAAGAG | TCAGCATCACAAAGAACTGAGA |
| 8 | *Niben261Chr08g0384017.1* | GAAATGATGGTGCCTCTCTACTC | CCATACTGTCACCTTCTGTTGT |
| 9 | *Niben261Chr12g0148002* | GAGGATTGGTGACTTCGGTT | GCAACAAACGCGGCAATA |
| 10 | *Niben261Chr06g0506004* | ACACAAGCACTGGCGTATT | TTGTACAGACCACCCAAAGTC |
| 11 | *Niben261Chr09g0038026* | GTTGACGGACGTAGTGGATAC | ACCTGCGTAGGAACAACAC |
| 12 | *Niben261Chr14g0906034* | TTCCACAGCCAATCCAAAGA | ATCTGAGGAATCTTGGCATAACA |
| 13 | *Niben261Chr08g1237018* | AGAAAGCAATCAGGAAGTAGAGG | AAATTGTCATCCTTCGCACAAG |
| 14 | *Niben261Chr14g0899022* | CCTCAAAGAATCGACCTTCAGT | ACTCACCCTCATAAACATCACC |
| 15 | *Niben261Chr16g0200004* | AATCCTTTGTCTCCAGCTTAGG | TAACAGCTTCAGGAGGTTGAAT |
| 16 | *Niben261Chr06g0166002* | GACTTCGGGATCCTGTTTATGG | CCGCAGTTTGGTACTTGGATAG |
| 17 | *CLCuMuV-CP* | GGCTTTGGTCAAGAAGTTTGTC | GCGTGGGTACAAGCCATATAA |
| 18 | *CLCuMuV-βC1* | TGATGAGCGATGGTGACTTG | TGACGAGGAGCAGAACAAAC |
| 19 | *PP2A* | GAGGGAGAGTGATTTGGTTGAT | CAGCTGATCTTCGCACCATA |
| 20 | *GAPDH* | GGGTGTCAACGAGAAGGAATAC | TTCCACCTCTCCAGTCCTT |
